# Multi-omics analysis and metastasis risk factor prediction in N1b stage PTMC: insights into immune infiltration and therapeutic implications

**DOI:** 10.3389/fimmu.2025.1620085

**Published:** 2025-09-03

**Authors:** Hao Dai, Qian Zhao, Wanli Ren, Qian Chen, Bei Pei, Wenyan Wang, Zhiqian Liu, Zhihan Liu, Jinzi Guo, Yuan Shao, Xiang Li, Yanxia Bai

**Affiliations:** ^1^ Department of Otolaryngology Head and Neck Surgery, The First Affiliated Hospital of Xi’an Jiaotong University, Xi’an, Shaanxi, China; ^2^ Center for Gut Microbiome Research, Med-X Institute Centre, The First Affiliated Hospital of Xi’an Jiaotong University, Xi’an, Shaanxi, China

**Keywords:** papillary thyroid microcarcinoma, immune infiltration, lymphatic metastasis, multiomics, machine learning algorithms

## Abstract

**Background:**

Papillary thyroid microcarcinoma (PTMC) with lateral neck lymph node involvement exhibits a deceptively indolent yet highly invasive phenotype, characterized by early dissemination and slow tumor growth. A comprehensive understanding of integrating multiomics landscapes, circulating immune profiles, and tumor immune microenvironment is essential for more accurate surveillance and tailored therapeutic strategies.

**Methods:**

Clinical profile and circulating immune-inflammatory markers from 638 PTMC patients were analyzed using multivariate and least absolute shrinkage and selection operator (LASSO) regression to recognize N1b-associated risk indicators. Eight supervised machine learning models were trained via 10-fold cross-validation to select the optimal classifier. Weighted gene coexpression network analysis (WGCNA) alongside machine learning identified metastasis-related gene modules from the integrated RNA-seq profile, leading to a multilayer perceptron gene classifier. Genomic profiling was employed to investigate mutations, copy number alterations, and methylation modifications in signature genes, followed by screening of antineoplastic drugs and docking simulations to explore their therapeutic potential. CIBERSORT, combined with immunohistochemistry, was used to investigate immune infiltration and functional changes in N1b-stage PTMC lesions.

**Results:**

Two clinical metastasis risk models were developed, with Model A based on the neutrophil-to-lymphocyte ratio (NLR) and Model B on lymphocyte and neutrophil counts, where Model A showed superior generalization (AUC = 0.852) and discriminative performance. NLR was an independent risk determinant for N1b-stage PTMC (OR = 2.12, p < 0.01). Transcriptomic profiling revealed a molecular signature (*ALDH1A3*, *CTXN1*, *MGAT3*, and *TMEM163*) of occult lateral lymph node metastasis, exhibiting strong robustness (AUC = 0.857). Signature genes were predominantly associated with cell adhesion, intercellular signaling, and KRAS dysregulation pathways. Hypomethylation of *CTXN1*, *MGAT3*, and *TMEM163* may underlie transcriptional activation. N1b-stage tumors exhibited reduced CD8+ T and T follicular helper cell infiltration but increased dendritic, *γδ* T, and activated CD4+ memory T cells, suggesting immune evasion and compensatory immune activation.

**Discussion:**

This study constructed a robust metastasis prediction nomogram for N1b-stage PTMC and identified metastasis-associated molecular drivers through integrative multiomics analysis. Comprehensive profiling of systemic and tumor-infiltrating immunity revealed key antitumor immune alterations. These findings establish a framework for early metastatic phenotype detection, potentially inspiring relevant immunotherapeutic hypotheses.

## 1 Introduction

Papillary thyroid carcinoma (PTC), the leading histological subtype of differentiated thyroid cancer (DTC), comprises approximately 84% of all thyroid carcinomas (1–3). The incidence of PTMC (maximum diameter ≤ 1 cm) has risen alongside advancements in ultrasound technology and increased health awareness. In the absence of lateral lymph node involvement (LLNM) and distant spread in most PTMCs, the latest American Thyroid Association (ATA) guidelines advocated active monitoring and the most focused intervention to reduce unnecessary surgical procedures (4–7). It is worth noting that invasive PTMCs develop LLNM at an early stage before tumour volume increases, and there are currently no specific clinical features or molecules that can reliably predict it.

Studies have explored the genomic landscape and ultrasonographic features of PTMCs, identifying numerous characteristics associated with the progression of high-risk subtypes, such as tumor vascularization and diffuse thyroid parenchymal involvement (8–12). The accurate diagnosis of LLNM remains challenging due to the limitations of preoperative fine-needle aspiration and the potential misinterpretation of intraoperative frozen sections of suspicious lymph nodes. Although clinical risk factors, such as capsular invasion and imaging scores from systems like TI-RADS, assist in assessing metastasis risk, their accuracy is constrained by the absence of multidimensional evidence, such as circulating immune-inflammatory markers. Hematological immune-inflammatory indicators, like lymphocyte-to-monocyte ratio (LMR) (13, 14), neutrophil-to-lymphocyte ratio (NLR) (15, 16), platelet-to-lymphocyte ratio (PLR) (17), and the systemic immune-inflammation index (SII) (18), are closely linked to the prognosis and recurrence of various cancers, including thyroid cancer. However, their association with LLNM in PTMC remains unclear. Therefore, this study seeks to combine clinical features with immune-inflammatory markers to develop a personalized model for improved clinical assessment of metastasis risk. Lateral lymph node metastasis is strongly associated with locoregional recurrence, distant metastasis, and unfavorable prognosis in PTMC, with *BRAF^V^* ^600^ *^E^* mutation further enhancing aggressiveness, resistance to radioactive iodine therapy, and recurrence risk (19, 20). In addition to well-established genetic alterations by next-generation sequencing in PTC, including *RET* rearrangements and mutation of *N/K/H-RAS*, and the *TERT* promoter region (C228T/C250T) (21, 22), there is a need for further exploration of the genomic and transcriptomic profiles of metastatic PTMC to identify potentially relevant factors closely associated with its progression. PTCs are characterized by a significantly heightened immune profile compared to normal thyroid tissue, with these changes closely linked to tumor progression. Compared to normal thyroid tissue, PTC patients exhibit higher levels of Th17 cells and dendritic cells (DCs) infiltration in tumor tissue (23). Additionally, elevated regulatory T cells (Tregs) and M0 macrophages are observed, and this trend correlates with higher T staging and lymph node metastasis. While natural killer (NK) cell infiltration is also enhanced, its degree of infiltration and functional activity decrease as the tumor progresses.

This study aims to identify metastasis risk factors in N1b-stage PTMC and to develop a highly accurate clinical model to facilitate clinical assessment. Additionally, we investigate the multi-omics landscape and immune infiltration profiles to provide molecular evidence for the supervision and intervention of early metastatic cases.

## 2 Materials and methods

### 2.1 Cohort selection and machine learning modeling

The retrospective study analyzed PTMC-confirmed patients who received initial surgical intervention at The First Affiliated Hospital of Xi’an Jiaotong University between January 2020 and January 2024. Cases with a history of other malignancies, recurrent disease, or secondary metastases requiring reoperation were excluded to minimize confounding factors. Demographic, histopathological, and hematologic parameters were retrospectively collected, including gender, age, pathological subtype, nodule count, lesion location, extrathyroidal extension (ETE), coexistence of Hashimoto’s thyroiditis (HT) or nodular goiter (NG), circulating counts of leukocytes (lymphocytes, neutrophils, and monocytes) and platelets, ratios including NLR, PLR, LMR, and SII, defined as the ratio of (neutrophil count × platelet count) to lymphocyte count. Complete data were available for all variables following the exclusion of records with missing values. To optimize feature selection, the least absolute shrinkage and selection operator (LASSO) regression was employed to identify variables significantly associated with aggressive clinicopathological behaviors. Subsequently, eight machine learning algorithms−AdaBoost, Logistic Regression, K-Nearest Neighbor (KNN), Decision Tree, Multilayer Perceptron (MLP), Extreme Gradient Boosting (XGBoost), Random Forest (RF), and Support Vector Machine (SVM) − were implemented to construct predictive models. Parallelly, univariate and multivariate logistic regression analyses were employed to uncover independent predictors, followed by the development of an alternative logistic regression model. Model validity was rigorously evaluated using evaluation criteria comprising area under the receiver operating characteristic curve (AUC), Brier score, accuracy, sensitivity, specificity, and F1 score. All reported metrics were derived from a 10-fold cross-validation procedure to enhance generalizability and avoid overfitting bias. The best-performing model was selected based on comparative performance across test cohorts, ensuring robustness and generalizability.

### 2.2 Collection of biospecimens and RNA sequencing

We collected tumor and corresponding adjacent thyroid tissue samples from six patients diagnosed with N1b-stage PTMC who underwent surgery in the Otolaryngology-Head and Neck Surgery department of our medical center from 2021 to 2022. All patients underwent thorough quality assessment and pathological diagnosis of PTMC by skilled pathologists. TNM classification and corresponding clinical staging were evaluated following the 8th edition AJCC/UICC staging system (24). We collected specimens from fresh ex vivo tissue with the premise of not compromising the pathological diagnosis. Consent was secured from all participants and/or their authorized representatives. All histological samples were flash-frozen in liquid nitrogen upon collection and preserved in the laboratory. Simultaneously, 30 N0-stage and 30 N1b-stage PTMC tissue section slides were retrospectively collected for subsequent immunohistochemical (IHC) analysis. Total RNA from the tumor specimens and their adjacent counterparts were separately isolated and purified using TRIzol (Invitrogen, Carlsbad, CA, USA). Count and Fragments Per Kilobase Million (FPKM) values for the N1b stage papillary thyroid microcarcinoma transcriptome atlas (PTMTA) were generated using StringTie (v.2.0.4) (25).

### 2.3 DEGs and construction of gene coexpression networks

Transcriptome microarray and clinical data for GSE129562 (26) and GSE153659 (27) were derived from the Gene Expression Omnibus (GEO) platform. RNA-seq and corresponding clinicopathological data for 25 PTMC-TCGA samples containing only N0 and N1b stages were acquired from the Xena platform following rigorous screening (28). All the selected samples were re-staged in accordance with the staging system of the AJCC (8th edition) and needed to fulfill the following exclusion criteria: (1) no history of prior thyroid surgery, (2) absence of any other malignant tumors, and (3) no comorbidities with other thyroid diseases. DEGs of the N1b-stage PTMC and adjacent tissues in the GSE129562 microarray dataset underwent screening via the “limma” (v.3.54.2) package (29) in R (v.4.2.0). Similarly, the DEGs of the PTMTA dataset were acquired by “DESeq2” (v.1.38.3) (30). The final DEGs were obtained by intersecting the aforementioned founded on the selection criteria FDR *<* 0.05 and log_2_ |FoldChange| *>* 1. The “clusterProfiler” (v.4.6.2) package facilitated the execution of GSEA (31), with sorted gene list files serving as input data. Microarray datasets of N1b and N0 stage PTMC samples were utilized for the development of coexpression networks via the weighted gene co-expression network analysis (WGCNA) algorithm and its corresponding R package (32). The clustering tree diagram was generated and the cut-off of tree height was set to 0.25 for merging similar modules.

### 2.4 Functional modules and candidate genes associated with metastasis

Next, we comprehensively analyzed the functional modules associated with LLNM. We calculated MEs as the principal components corresponding to each module. The relationship between MEs and N staging was analyzed with Pearson’s correlation coefficient. A module that exhibited a significant correlation between gene significance (GS) and module membership (MM) was considered to be highly associated with metastasis. We separately submitted the designated modules to the DAVID platform (https://david.ncifcrf.gov/) (33) for enrichment terms of GO functional classification and Kyoto Encyclopedia of Genes and Genomes (KEGG) pathways, including BP, MF, and CC. The leading 10 terms meeting criteria of count ≥ 4 and *p <* 0.05 were discussed and visualized. Candidate genes, determined based on their intramodular connectivity and correlation with tumor metastasis, play pivotal roles within the modules and exhibit substantial interconnectivity within the coexpression network, satisfying the specified criterion of |MM| *>* 0.8 and |GS| *>* 0.2. Subsequently, genes within the selected modules were submitted to the STRING online platform and developed protein-protein interaction (PPI) networks. The molecular complex detection (MCODE) of Cytoscape (v.3.7.2) was then utilized to identify the core subnetworks. The intersection between the core genes of specific modules and genes within the PPIs network was identified as the set of candidate genes, which were further analyzed and validated. Thereafter, we obtained differentially expressed candidate genes by taking the intersection with the DEGs previously obtained.

### 2.5 Consensus clustering of candidate markers

Based on the transcriptomic matrix of the merged PTMC training set, molecular subtypes were established through unsupervised consensus clustering of 10 differential candidate genes with the “ConsensusClusterPlus” package (v.1.62.0). The cluster count (k) was capped at six, and 1000 subsampling iterations were performed to ensure the robustness. Consequently, the optimal k value was finally identified by the CDF plot, the consensus matrix heatmap, and the beneath under the CDF curve. Additionally, a heatmap was generated to illustrate the differences in the transcriptome for candidate biomarkers across different subtypes. Furthermore, unsupervised clustering of 458 large-volume PTC transcriptomic matrices from the TCGA database was performed using the above candidate genes to further explore their respective correlations with various clinical characteristics. Samples with T1-T2 stages combined with N1b were categorized as high lymph node metastatic risk (LNM-high), while those with T3-T4 stages combined with N0 were designated as low lymph node metastatic risk (LNM-low).

### 2.6 Machine learning-based molecular metastasis model

The PTMTA, GSE129562, and PTMC-TCGA datasets were integrated, and 25 N0 and 14 N1b-stage PTMCs were selected as the training set. RF and support vector machine recursive feature elimination (SVM-RFE) algorithms were utilized to further filter biomarkers closely associated with metastasis. The candidate genes were prioritized based on their ranking of relative importance using the “RandomForest” package (v.4.7-1.1) (34), and gene importance greater than 0.5 and ranked within the top 5 were designated as significant biomarkers. The “e1071” and “caret” (v.4.7-1.1) packages were utilized for training the feature subset of the expression matrix via the SVM-RFE algorithm. Utilizing 5-fold cross-validation, the points with the lowest error rate and the highest accuracy rate were determined, and the top 5 biomarkers were selected as feature genes. Ultimately, the overlapping of the feature genes screened through the above algorithms yielded biomarkers highly associated with LLNM. The “neuralnet” (v.1.44.2) package was utilized to construct an MLP identifier for prediction and diagnosis in the artificial neural network (ANN) framework. The area under the curve (AUC) serving as a metric for assessing the discriminative power of the MLP classifier. Additionally, we obtained the AUC on a separate test cohort (GSE153659) to further evaluate the generalizability of the model.

### 2.7 Functional analysis of GSEA and genomic characteristics

The required gene sets for this study were sourced from the GSEA platform (https://www.gsea-msigdb.org/gsea/msigdb/index.jsp). The GSEA function was used to enrich biological functions associated with a single gene. Functional scores for each sample within the training cohort were computed using the “GSVA” (v1.46.0) package (35), enabling the assessment of the absolute enrichment of corresponding gene sets. Furthermore, differences in enrichment across different functional pathways between the N0 and N1b groups were compared. Mutation, copy number alterations, and methylation (HM450) matrices for each signature gene were obtained from TCGA-THCA via the cBioPortal (https://www.cbioportal.org/datasets). Following the filtration of PTC tumor tissue samples to focus on genomic alterations within the signature genes, we conducted a comprehensive investigation of the correlation between gene methylation and transcription levels. This allowed us to gain deeper insights into the genomic mechanisms driving transcriptional regulation and better understand the factors influencing gene expression in PTC.

### 2.8 Clinical variations of signature genes and IHC

To investigate the clinical relevance of metastasis-associated signature genes, we analyzed PTC tumor sample matrices for N0 and N1 stages obtained from the TCGA database. Expression profiles of these signature genes were assessed across various clinical factors such as age groups and TNM staging, with significant variations identified. Furthermore, stratified analysis by age was conducted to further investigate the transcriptional differences of signature genes within various clinical stages, offering insights into their role across different disease progressions. Moreover, IHC validation was conducted on 30 N0-stage and 30 N1b-stage PTMC tissue section slides. All pathological slides underwent independent evaluation by three skilled pathologists and complied with the following (a) the positive intensity score (no staining, 0; light yellow, 1; tan-yellow, 2; brown, 3) and (b) the positive area score (0, *<*5%; 1, 5%-25%; 2, 26%-50%; 3, 51%-75%; 4, *>*75%). Ultimately, the immunoreactivity score (IRS) was calculated by multiplying the two indicators above. The consistency of IRS evaluations by three independent pathologists was quantified using intraclass correlation coefficients (ICCs). ICCs were computed based on a two-way mixed-effects model with average rating measurements, implemented in SPSS version 22.

### 2.9 Drug response profiling and target docking

Tumor sensitivity to anticancer drugs in papillary thyroid microcarcinoma (PTMC) was predicted using Ridge regression models implemented via the R package “oncoPredict”, with drug response information derived from the Genomics of Drug Sensitivity in Cancer (GDSC) database. Predicted IC_50_ values were compared across clinical subgroups to identify differential drug sensitivities. Molecular docking procedures employed AutoDock Vina, and the optimal post-docking conformation was selected through a comprehensive evaluation of binding free energy (docking score), conformational rationality, and interaction forces. The protein crystal structures were retrieved from the RCSB Protein Data Bank (PDB; https://www.rcsb.org), while the three-dimensional structures of small-molecule compounds were acquired from the PubChem database (https://pubchem.ncbi.nlm.nih.gov/).

### 2.10 Immune infiltration profile

We quantitatively evaluated the abundance of infiltrating immune cell subsets in the PTMC tissue specimens of the N0 and N1b groups through the “CIBERSORT” algorithm(v.0.1.0) (36), with the LM22 reference matrix, which is specifically designed to distinguish 22 human immune cell phenotypes based on gene expression profiles. The results are visually presented in stacked bar plots, and the variance in immune cell infiltration across the two groups was assessed through *t*-tests. The “corplot” (v.0.92) package was employed to analyze the correlations among the immune cell subtypes and generate a heatmap. To evaluate immune functional differences between the metastatic and non-metastatic PTMCs, single-sample Gene Set Enrichment Analysis (ssGSEA) was performed using a curated panel of 13 immune-related gene sets. These gene sets were compiled from published studies (37) and the MSigDB database. Normalized enrichment scores (NES) were calculated for each sample based on the PTMCs matrix. Intergroup variation in immune function was analyzed using the non-parametric Wilcoxon rank-sum test to determine statistical significance. Immunohistochemical analysis was conducted on primary tumor specimens from 20 N1b-stage and 20 N0-stage PTMC patients to delineate the differential infiltration patterns of key immune cell populations. Moreover, an assessment was carried out to explore the connection between signature biomarkers and the 13 predefined immune-related functions.

### 2.11 Statistical analysis

Clinical modeling and bioinformatics analysis workflows in this research were implemented via R software (v.4.2.0) and corresponding packages. The cohort underwent random allocation into training (70%) and test (30%) sets via stratified sampling, ensuring comparability in key clinical variables. Eight models−AdaBoost, logistic regression, random forest, decision tree, XGBoost, SVM, KNN, and MLP−were trained using 10-fold cross-validation to optimize hyperparameters and ensure generalizability. Clinical utility was evaluated via decision curve analysis (DCA), while calibration curves with bootstrapping quantified prediction-reality alignment. Concurrently, variables without multicollinearity (VIF*<*5) underwent univariate (p*<*0.10) and multivariable (p*<*0.05) regression to develop a secondary clinical nomogram, with comparisons between the two models conducted based on net reclassification improvement (NRI) and integrated discrimination improvement (IDI). The Wilcoxon rank-sum test was employed to evaluate differences in proportions of infiltrating immune cells and immune functionality. Comparative analysis of IRSs was completed using GraphPad Prism (v.8.0.2, San Diego, California, USA).

## 3 Results

### 3.1 Clinical feature selection via regularized regression

**Figure 1:**
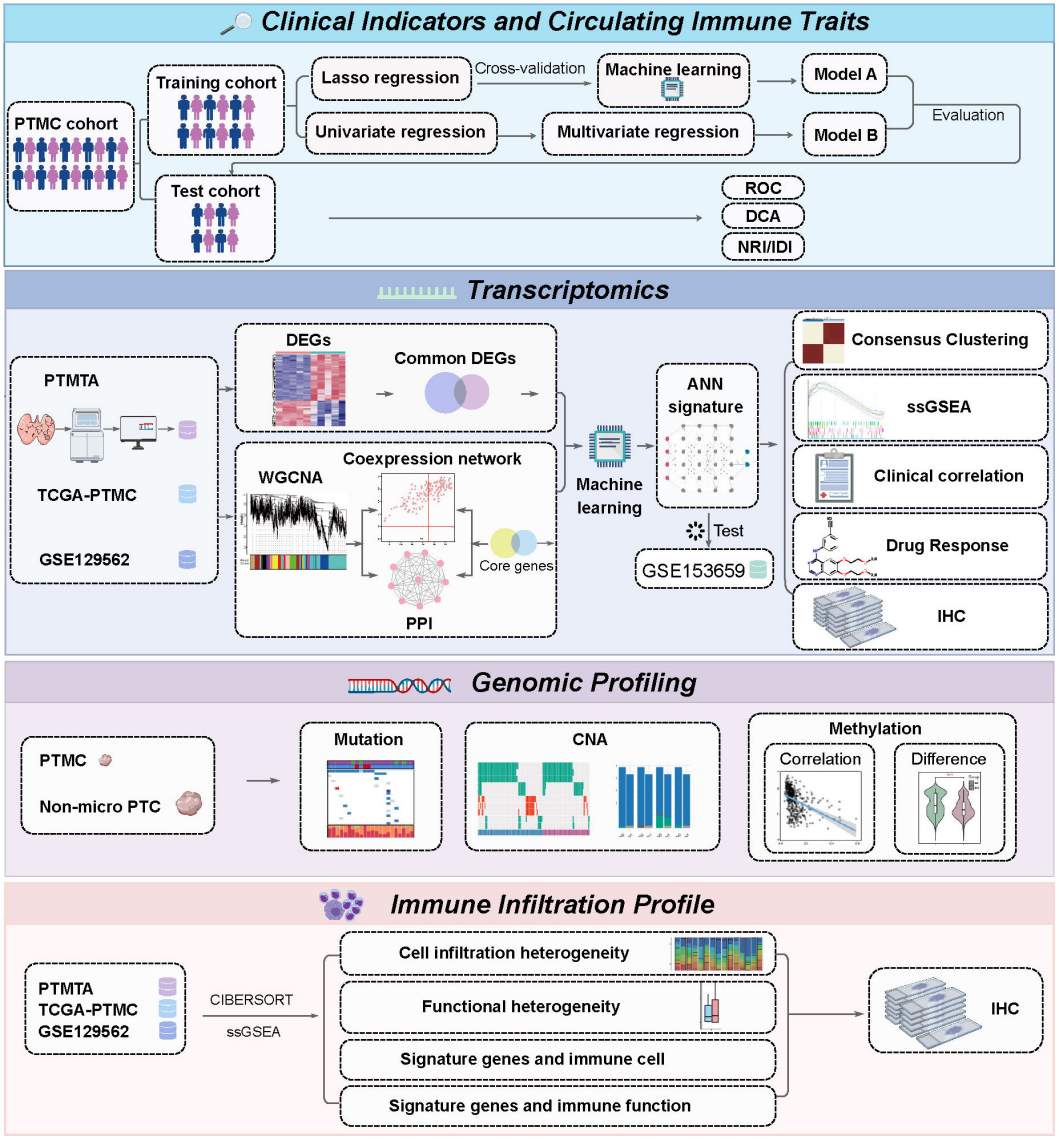
Schematic overview of the analytical workflow.

**Figure 2:**
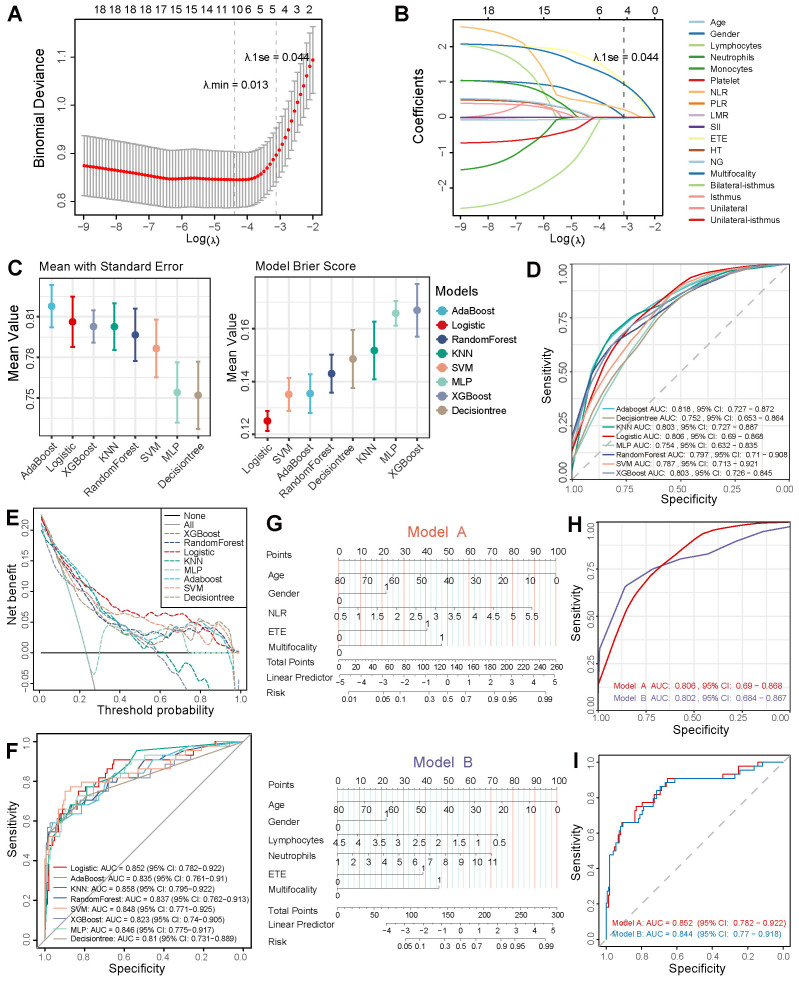
Feature screening and machine learning modeling. **(A, B)** LASSO regression for feature selection. **(C)** AUC and Brier scores of eight machine learning models. **(D)** Mean ROC curves from 10-fold cross-validation on the training cohort. **(E, F)** Clinical decision curves and ROC curves of eight models on the independent test cohort. **(G)** Nomograms of Model A (LASSO-derived) *vs.* Model B (multivariate regression-based). ROC curves validated via 10-fold cross-validation across training **(H)** and independent test cohorts **(I)**.

The layout of the study design is outlined in **Figure 1**. The study cohort comprised 638 patients, with approximately 23.2% of cases exhibiting lateral neck lymph node metastasis, as shown in (**Supplementary Table 1**). The demographic characteristics of the randomly allocated training (n = 447) and testing (n = 191) cohorts demonstrate no statistically significant intergroup differences in baseline features. LASSO regression with 10-fold cross-validation identified five predictive predictors with non-zero coefficients at _λ_.1se: age, gender, NLR, ETE, and multifocality (**Figures 2A, B**). Subsequent comparative analysis of eight machine learning models revealed the logistic regression model as the optimal classifier, exhibiting superior discriminative performance (AUC: 0.806), enhanced calibration accuracy (Brier score: 0.125), and greater clinical utility across threshold probabilities in decision curve analysis compared to alternative approaches (**Figures 2C–F**) (**Supplementary Table 2**). Comparative validation between the LASSO-derived Model A (incorporating age, gender, NLR, ETE, and multifocality) and multifactorial Model B (age, gender, lymphocyte counts, neutrophil counts, ETE, and multifocality) demonstrated Model A’s consistent superiority. In 10-fold cross-validated training evaluations, Model A achieved an AUC of 0.806 (95% CI: 0.69 - 0. 868) versus Model B’s 0.802 (95% CI: 0.684 - 0.867) in training cohort (**Supplementary Table 3**), with this performance advantage maintained in independent testing (AUC: 0.852 *vs.* 0.844) (**Figures 2G–I**). Model B exhibited significantly poorer reclassification performance (NRI = -0.338, IDI = -0.0081; both *p* ≤ 0.05), underscoring Model A’s threshold-agnostic superiority in risk stratification accuracy.

### 3.2 Detection of DEGs and GSEA

**Figure 3:**
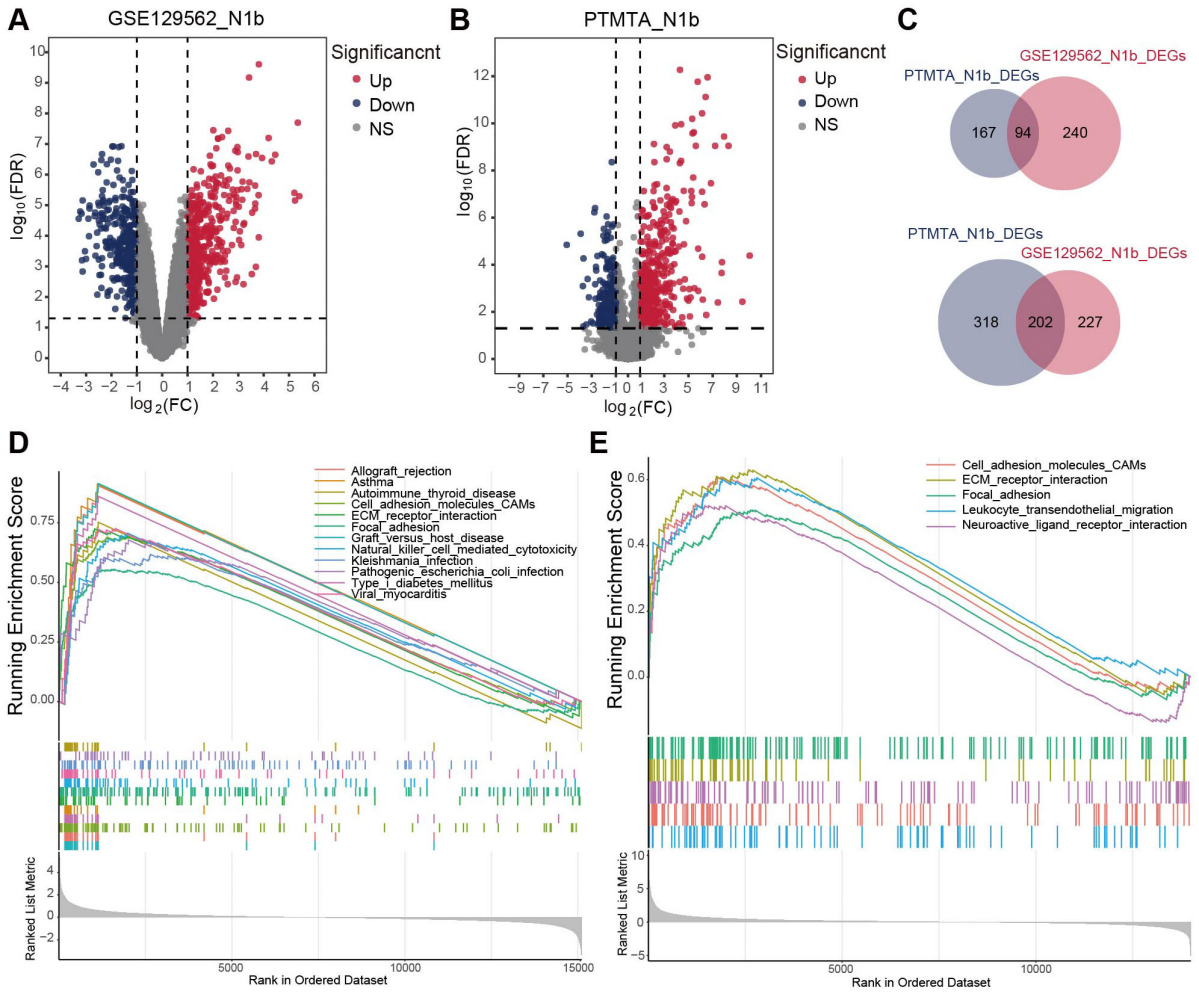
The transcriptome landscape of N1b stage PTMC and paired adjacent thyroid tissue. **(A, B)** Volcano diagrams illustrating DEGs within the GSE129562 and PTMTA cohorts. **(C)** The Venn diagrams depicted an overlap of 94 upregulated DEGs and 202 downregulated DEGs in both cohorts. GSEA terms for N1b stage samples in the **(D)** GSE129562 and **(E)** PTMTA cohorts.

Six pairs of solitary N1b-stage PTMC tissues and their corresponding adjacent thyroid tissues were selected for RNA sequencing, resulting in a resource known as the Papillary Thyroid Microcarcinoma Transcriptome Atlas (PTMTA) (**Supplementary Table 4**). RIN scores (8.2-9.4) confirmed that all RNA samples met quality standards required for transcriptomic applications. The raw data were subjected to background correction and normalization (**Supplementary Figures 1A, B**). To screen for differentially expressed genes (DEGs) between N1b stage PTMC tissues and adjacent nontumor tissues, we separately acquired them from PTMTA and the GSE129562 microarray data (containing five pairs of N1b samples) and subsequently cross-merged to obtain the intersection (**Figures 3A, B**). 296 genes exhibited significant differential expression, with 202 genes being downregulated and 94 genes being upregulated (**Figure 3C**). Representative heatmaps of the overlapping genes in the GSE129562 and PTMTA datasets were generated (**Supplementary Figures 1C, D**). The DEGs between N1b- and N0-stage PTMC within the GSE129562 dataset are shown in **Supplementary Figure 1E**. Gene set enrichment analysis (GSEA) terms revealed common significant enrichment of cellular adhesion molecules, extracellular matrix (ECM) receptor interactions, and focal adhesion activation in both datasets (**Figures 3D, E**).

### 3.3 Weighted coexpression network and module screening

**Figure 4:**
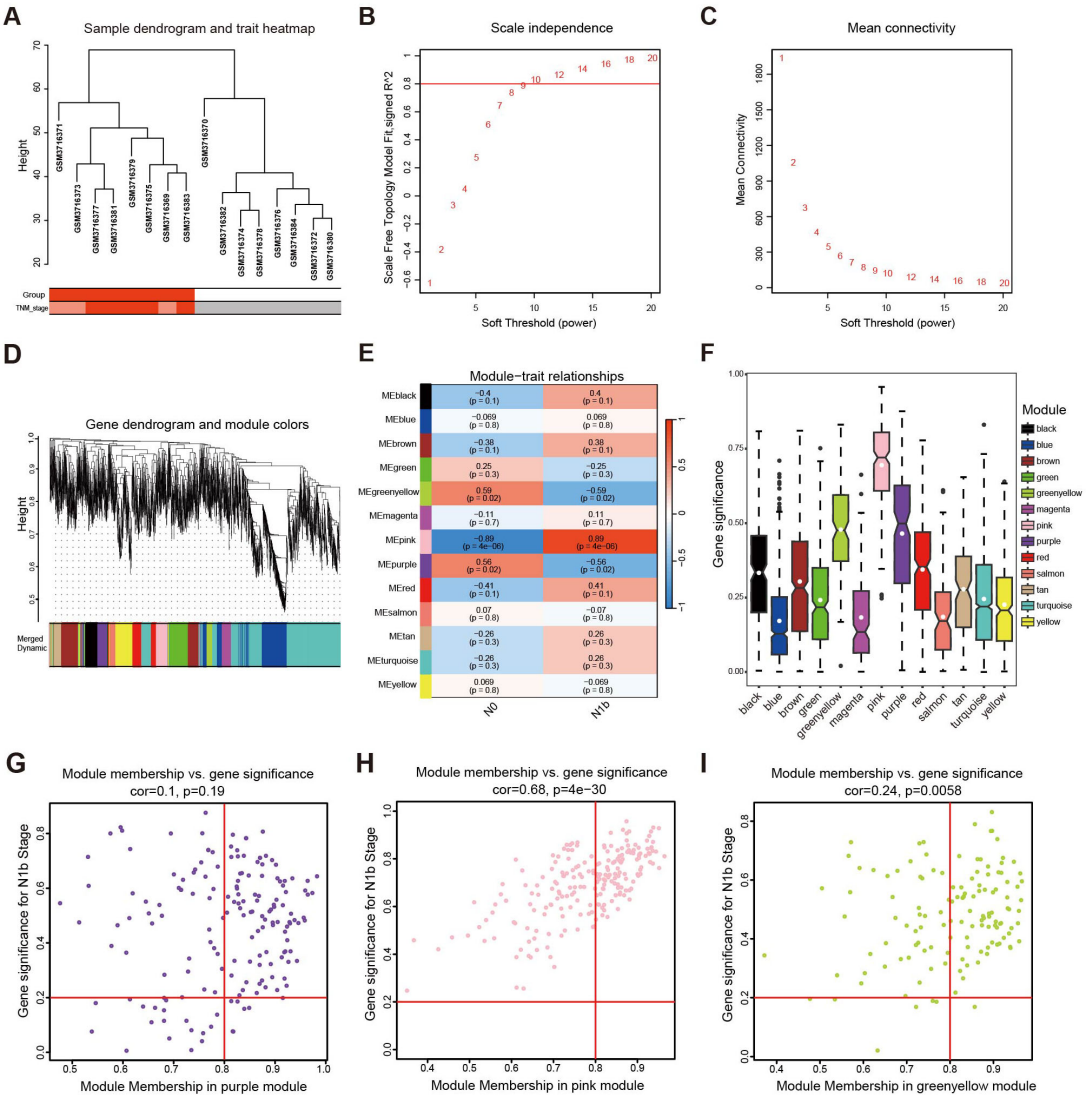
Development of a weighted coexpression network and module screening. **(A)** Sample clustering tree diagram to identify outlier cases. **(B)** The scale-free fit measure (*R* ^2^) with the corresponding soft threshold value (*β*). **(C)** Mean connectivity corresponds to each soft threshold power. **(D)** Hierarchical clustering tree: the modules with a dissimilarity lower than 0.25 were merged. **(E)** Heatmap illustrating the correlation between the modules and the pathological stage. **(F)** Mean gene significance and error plots of different modules. **(G)** Scatter plots of genes characterizing the purple module, **(H)** pink module, and **(I)** green-yellow module based on the selection criteria of |MM| *>* 0.8 and |GS| *>* 0.2 denoted by red lines.

After clustering the samples to detect any outliers (**Figure 4A**), we selected values from 1 to 20 for network topology analysis of the samples. Upon setting the optimal soft threshold to 9, we obtained a scale-free topology fit index (*R* ^2^) of 0.8, indicating a relatively even distribution of scale independence and a higher level of average connectivity (**Figures 4B, C**). Then, we constructed scale-free networks and topology matrices, plotted hierarchical clustering trees, and merged module maps, resulting in 13 modules for further analysis (**Figure 4D**). By assessing the correlation between module eigengenes (MEs) and LLNM, we observed that pink module demonstrated a notable positive correlation with LLNM (*r* = 0.89, *p* = 4 × 10^−0.6^). In contrast, the green-yellow module (*r* = −0.59, *p* = 0.02) and the purple module (*r* = −0.56, *p* = 0.02) exhibited a negative correlation with metastasis (**Figure 4E**). Additionally, upon further examination, it was found that the pink and green-yellow modules had greater absolute GS values (**Figure 4F**), and their MM *vs.* GS scatter plots indicated a strong correlation with metastasis (*p* ≤ 0.05) (**Figures 4G–I**).

### 3.4 Functional enrichment and biomarker screening

Enrichment analyses were carried out on the pink and green-yellow modules. Gene ontology (GO) terms for the pink module indicated significant involvement of candidate genes in the negative regulation of cell proliferation and cycle, while protein products were prominently involved in forming cytoplasm, plasma membrane and extracellular exosome, and protein binding being the most highly enriched molecular function (MF) (**Supplementary Figures 2A–C**). Notably, the KEGG-enriched terms of the pink module were metabolic pathways (**Supplementary Figure 2D**). For the green-yellow module, candidate genes exhibited primary enrichment in biological processes including cell adhesion, O-glycan processing, and small GTPase-mediated signal transduction regulation, and their products are primarily associated with integral components of the membrane (**Supplementary Figures 2E, F**). The predominant MFs of this module were protein homodimerization and microtubule binding, while the associated genes were linked to ECM-receptor interaction and mucin-type O-glycan biosynthesis pathways (**Supplementary Figures 2G, H**).

**Figure 5:**
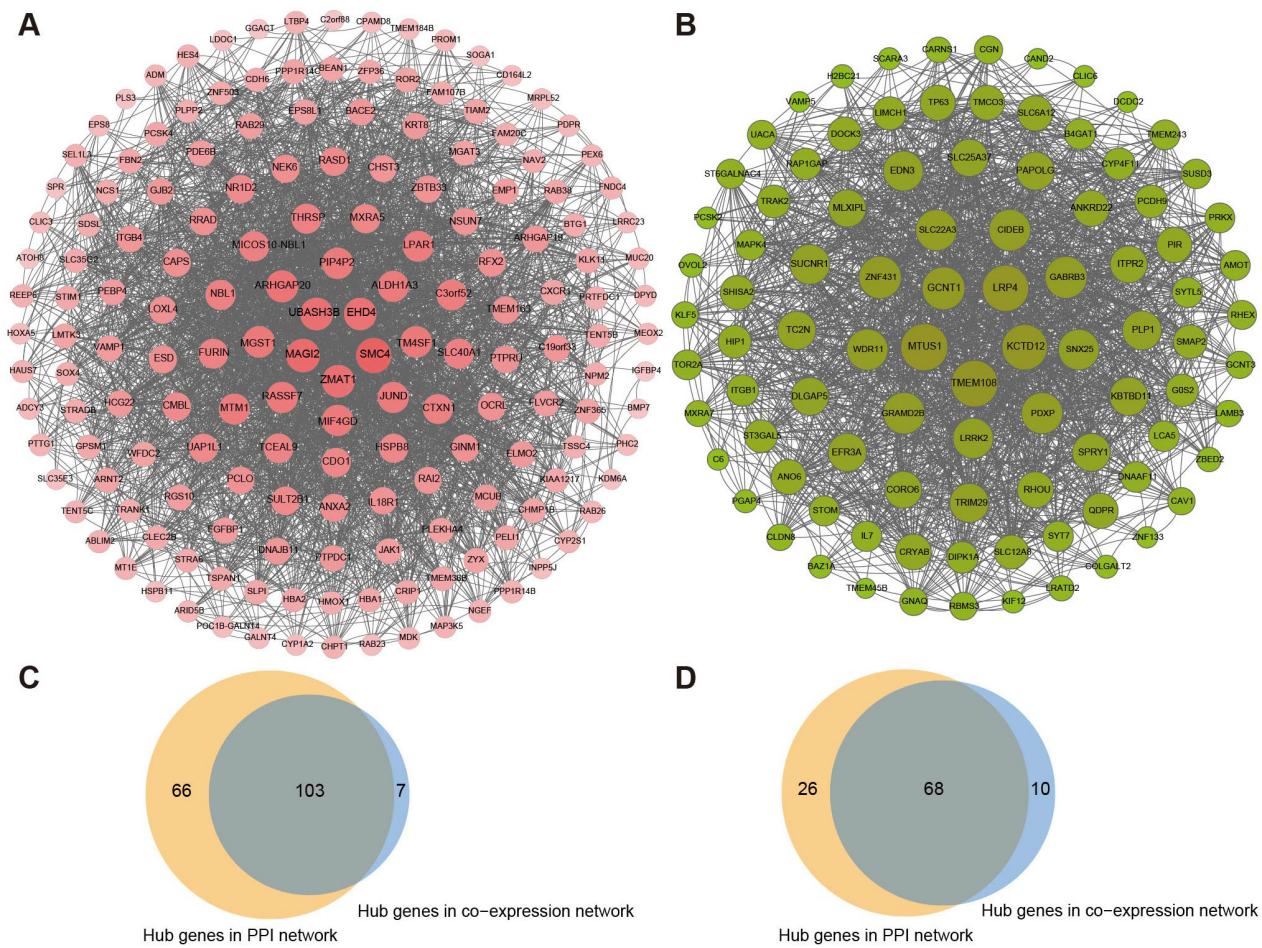
Generating PPI networks and filtering of critical biomarkers. The PPI networks of **(A)** the pink module and **(B)** the green-yellow module were filtered by the MCODE algorithm. **(C)** Screening of overlapping genes of the coexpression and PPI networks in the pink module and **(D)** green-yellow module.

In PPI networks, 169 biomarkers of the pink section and 94 biomarkers of the green-yellow section were subsequently analyzed as the core genes using MCODE (**Figures 5A, B**). Based on the cut-off value in WGCNA, 110 biomarkers in the pink section and 79 markers in the green-yellow section were recognized as highly related to metastasis. Moreover, the intersection of the above results showed that 103 genes of the pink section and 68 genes of the green-yellow section were filtered as candidate biomarkers associated with LLNM (**Figures 5C, D**). Subsequently, 11 biomarkers in the pink section and 6 biomarkers in the greenyellow section were filtered by transcriptional profiling comparison of the candidate genes in N1b stage PTMCs. The expression of the above markers was further validated in the PTMC-TCGA and GSE153659 cohorts (**Supplementary Figure 3**, **Supplementary Table 5**). Ultimately, six genes (*ALDH1A3*, *CDH6*, *CTXN1*, *HBA1*, *MGAT3*, and *TMEM163*) from the pink module and four genes (*LRP4*, *LRRK2*, *MAPK*, and *SNX25*) from the yellow-green module exhibited major differential expression and were found to be associated with LLNM.

### 3.5 Cluster validation of molecular subtypes

**Figure 6:**
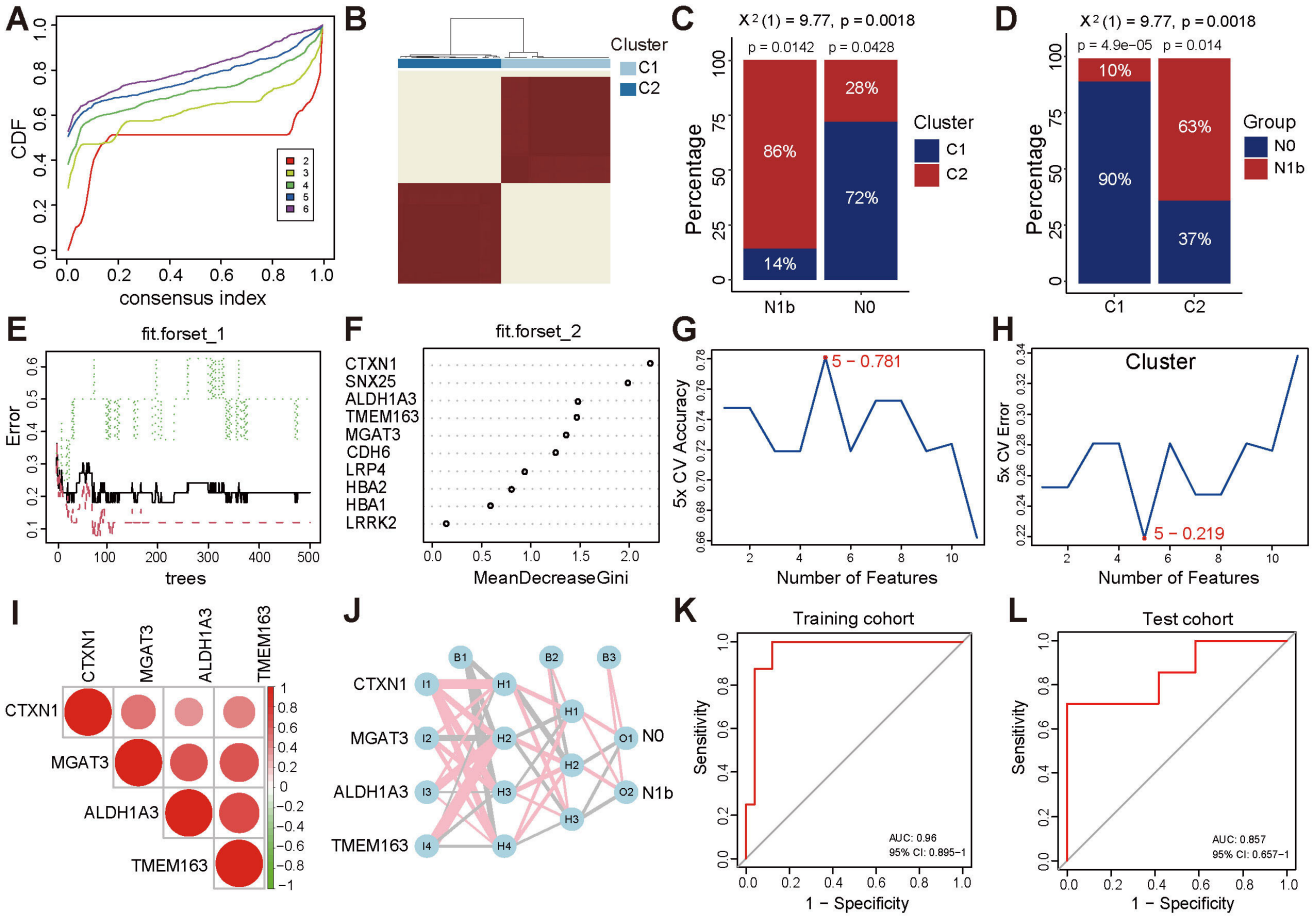
Consensus-based molecular subtyping and evaluation of gene signature performance. **(A)** CDF trajectory plot for consensus clustering (k = 2 - 6). **(B)** Consensus matrix plot (k = 2). **(C)** Distribution of N0 and N1b stage patients across the C1 and C2 clusters in the PTMC cohort. **(D)** Proportion of patients with varying N stages within the C1 and C2 clusters. **(E)** Diagram of the classification decision tree constructed by the RF algorithm. **(F)** Relative importance ranking. **(G, H)** The SVM-RFE algorithm screened the top 5 genes with accuracy and error rate. **(I)** Heatmap of correlations between signature genes. **(J)** MLP identifier of signature genes. **(K, L)** ROC curve of the MLP classifier in the training cohort and the separate cohort.

To further validate the discriminative ability of 10 differential candidate biomarkers concerning lateral cervical lymph node involvement in PTMC patients, unsupervised clustering of specific gene expression profiles was analyzed in the training set, comprising the merged PTMC cohorts (14 N1b *vs.* 15 N0). The optimal number of clusters (k) was 2 (clusters 1 and 2) based on the alterations in the area beneath the cumulative distribution function (CDF) curve, consensus CDF trajectory curve, and the consensus clustering heatmap (**Figures 6A, B**, **Supplementary Figures 4A, B**). Moreover, the heatmap for clusters revealed distinct characteristics between the two subgroups (**Supplementary Figure 4C**). Notably, patients with a low risk of lymph node metastasis were primarily concentrated in cluster 1 (72%, *p* ≤ 0.05), whereas the C2 cluster group exhibited a higher rate of lymph node metastasis (63%, *p* ≤ 0.05, **Figures 6C, D**). The same methodology was applied to the large-volume PTC from the TCGA-THCA cohort, yielding similarly well-differentiated clusters (**Supplementary Figures 4D–J**).

### 3.6 Metastasis-related signature genes

The RF algorithm integrated the number of classifications with the error rate in the decision tree for modeling (**Figure 6E**). The top five genes were determined based on the MeanDecreaseGini metric, where a higher value indicates greater importance (**Figure 6F**). Moreover, the SVM-RFE method selected the top 5 genes that exhibited the optimal accuracy rate (**Figure 6G**) and the minimum error rate (**Figure 6H**). By taking the intersection of the results mentioned above, the top 4 biomarkers, *ALDH1A3*, *CTXN1*, *TMEM163*, and *MGAT3*, were determined to be signature genes associated with metastasis. Additionally, the positive correlation between signature genes indicated a similar biological functional trend (**Figure 6I**). The MLP identifier was established utilizing the expression landscape consisting of signature genes from the training set. Through rigorous 5-fold cross-validation training, a robust MLP identifier was successfully developed (**Figure 6J**) and demonstrated notable performance in the training set, achieving an AUC of 0.96 (**Figure 6K**). Furthermore, subsequent evaluations illustrated its good generalization ability, as evidenced by an AUC of 0.857 for N0 and N1 stage PTMC patients in the independent test cohort GSE153659 (**Figure 6L**).

### 3.7 GSEA profiling

**Figure 7:**
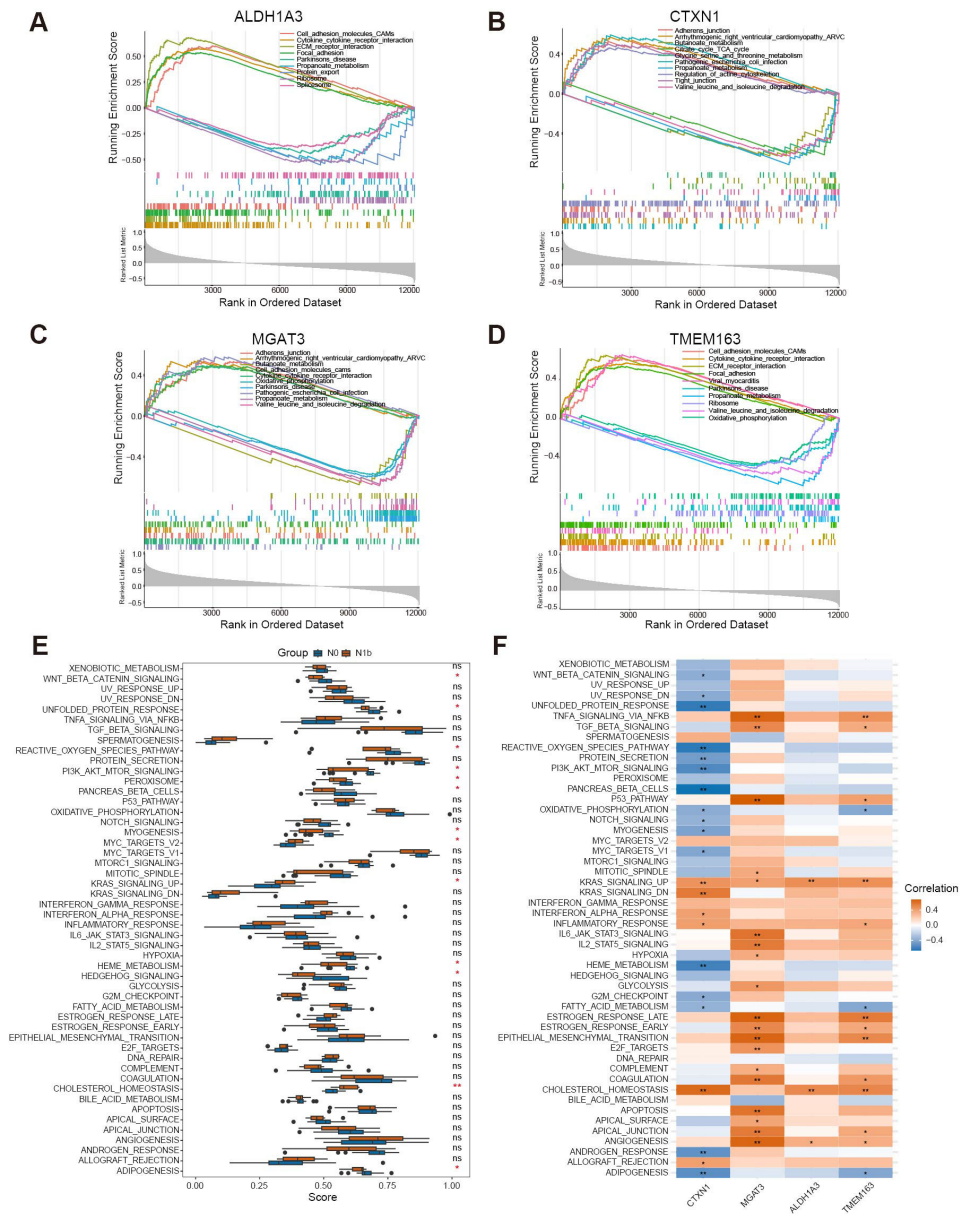
Single-gene GSEA and ssGSEA enrichment terms of signature biomarkers. **(A–D)** SgGSEA terms revealed the top 5 upregulated and downregulated functional pathways of the signature genes. **(E)** Boxplot illustrated the differential functional ssGSEA scores of signature genes. **(F)** Heatmap depicted the relationship between signature genes and primary hallmark functions (^∗^ *p <* 0.05, ^∗∗^ *p <* 0.01, ns means non-significant).

To elucidate the molecular changes underlying the progression of PTMC and to comprehensively investigate the biological functions in connection with signature genes, the gene expression patterns of the four genes were further analyzed across various biological processes using single-gene gene set enrichment analysis (sgGSEA) (**Figures 7A–D**). *MGAT3* and *CTXN1* were significantly enriched in adhesion junctions. *ALDH1A3*, *TMEM163*, and *MGAT3* exhibited significant association with cytokine-cytokine receptor interactions and the function of cell adhesion molecules. Notably, high expression of *ALDH1A3* and *TMEM163* was also observed to promote ECM receptor interaction and focal adhesion. *ALDH1A3* and *CTXN1* seem to be involved in regulating the actin cytoskeleton. Additionally, *CTXN1* was enriched in the tight junction pathway, suggesting its potential involvement in pivotal biological processes, including establishing intercellular tight junctions and preserving cellular polarity. Notably, the four signature genes displayed negative associations with diverse metabolic pathways.

By performing ssGSEA on hallmark gene sets, we achieved enhanced insights into the functional disparities among distinct stages of PTMC. Remarkably, the cohort with lymph node metastasis exhibited a significant increase in the function of KRAS signaling, cholesterol homeostasis, and MYC target v2. Conversely, the functions of the Wnt/*β*-catenin pathway, PI3K/AKT/mTOR pathway, and Hedgehog pathway markedly decreased in the N1b group (**Figure 7E**). Furthermore, a comprehensive analysis revealed significant relationships between the signature genes and the biological functions represented by the hallmark gene sets (**Figure 7F**). These findings reveal that signature genes may affect the abovementioned potentially dysregulated molecular pathways and metabolic processes during the dissemination of tumor cells to regional lymph nodes.

### 3.8 Mutations, CNAs, and methylation of signature genes

**Figure 8:**
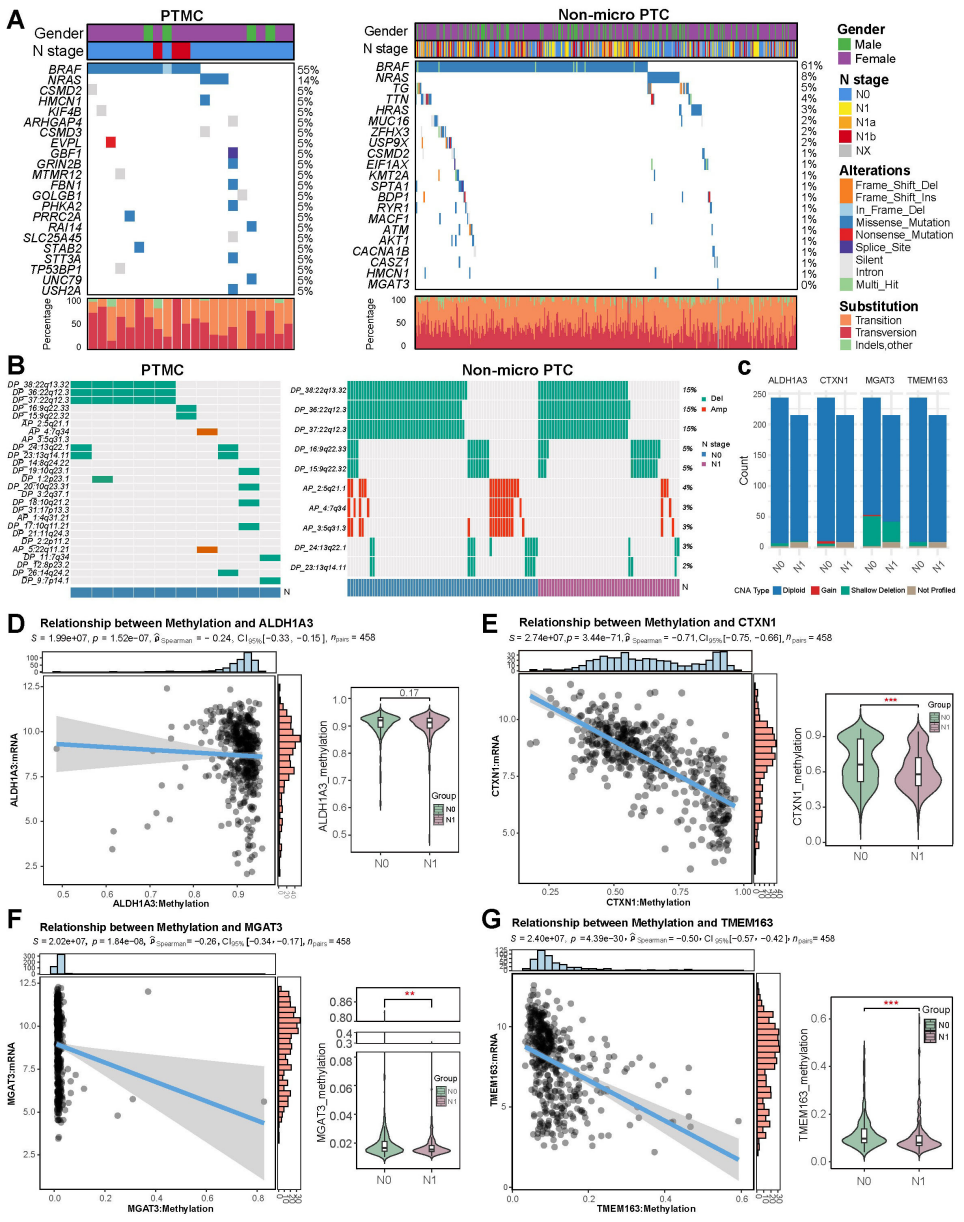
Integrated genomic and epigenetic landscape of PTMC and non-micro PTC. **(A)** Somatic mutation profiles. **(B)** Heatmap of CNAs. **(C)** Comparison of CNA profiles for signature genes across subgroups. **(D–G)** Methylation-mediated regulation and N stage-specific methylation differences of signature genes.

Comparison of mutation spectra between 22 PTMC and 412 non-micro PTC tumors revealed *BRAF* and *NRAS* mutations as predominant driver alterations in both groups (**Figure 8A**). *HRAS* mutations were notably more prevalent in the non-micro PTC cohort. Although mutations in *TG* and *TTN* genes occurred at relatively higher frequencies, the low proportion of functionally relevant missense variants suggests these are unlikely to play major oncogenic roles. A single N0-stage non-micro PTC case harbored the *MGAT3* G16D mutation, while no mutations were observed in the remaining signature genes. Additionally, no significant differences in CNA profiles were identified between the 10 PTMC and 414 non-micro PTC cases, with deletions most frequently observed on chromosomes 22q and 9q, and amplifications primarily occurring on 5q and 7q (**Figure 8B**). Further exploration into the genomic alterations of the four signature markers in 458 TCGA-PTC tissue revealed copy number amplification of *CTXN1* and *MGAT3* based on DNA-seq data (**Figure 8C**). In contrast, genomic landscape analysis revealed no group-specific patterns in CNAs of the four signature genes, as assessed by Fisher’s exact test. Moreover, while shallow deletions were sporadically detected in the four signature genes, their overall influence on genetic transcription appeared to be minimal. We assessed the methylation patterns of four genes across various clinical subgroups and observed that patients in stage IV exhibited significantly reduced methylation of genes *ALDH1A3* and *TMEM163* compared to those in stages I−II, while patients with lymph node metastasis showed a decrease in methylation of *CTXN1*, *MGAT3*, and *TMEM163* (**Figures 8D–G**), suggesting that the hypomethylation-induced transcriptional activation of these genes may contribute to tumor metastasis and the progression to advanced stages. Additionally, the methylation status of *CTXN1* and *TMEM163* varied significantly across different T stages and age groups, suggesting stage- and age-dependent epigenetic regulation (**Supplementary Figure 5**).

### 3.9 Clinical stratification and IHC

**Figure 9:**
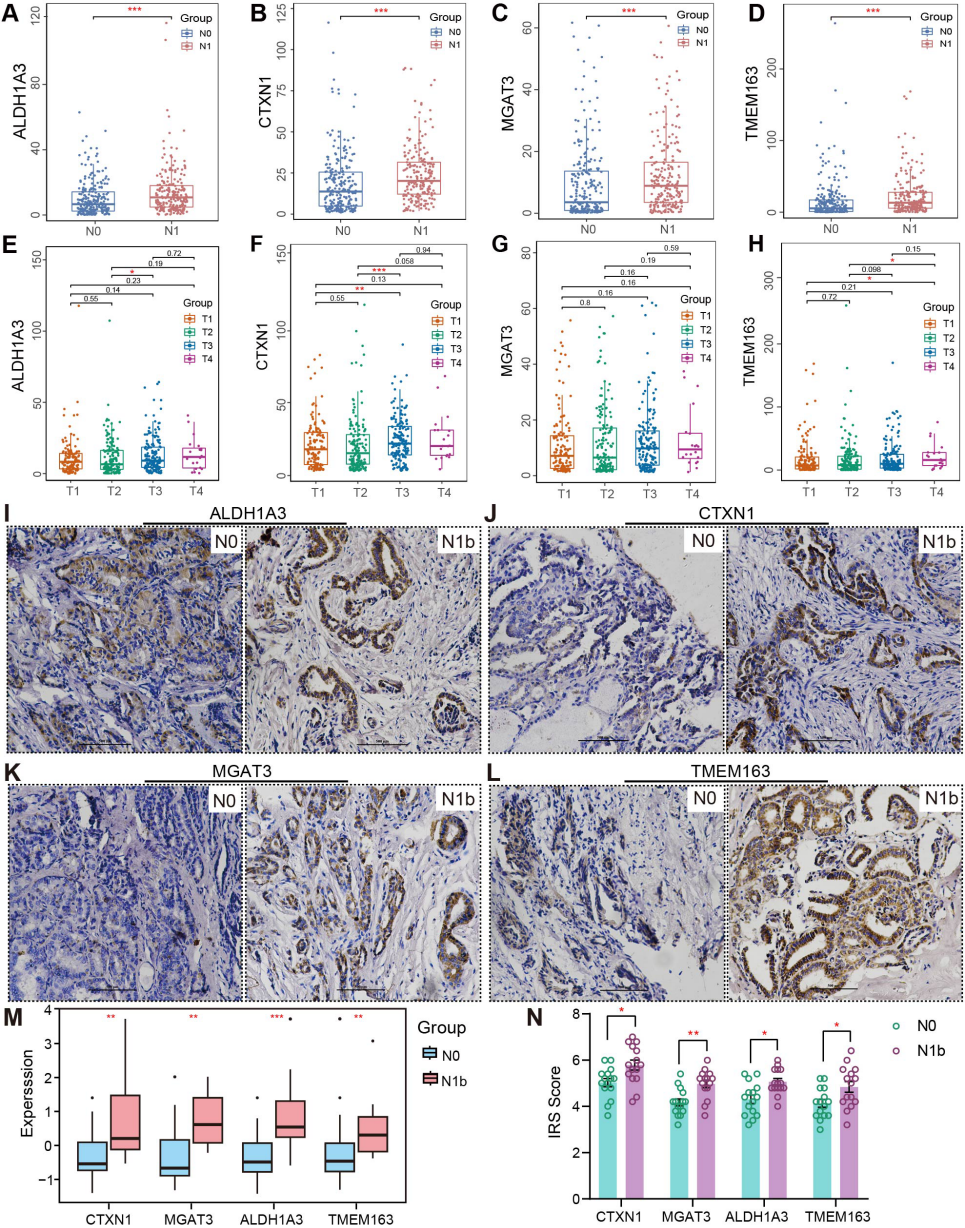
Clinical subgroup differences and IHC validation. **(A–D)** RNA discrepancy across various N stages and **(E–H)** T stages. **(I–L)** Representative immunohistochemical images of PTMCs in the N1b and N0 groups (20×). Scale bar, 500 µm. **(M)** Expression intensity analysis of signature genes in the merged PTMC cohort. **(N)** IHC staining results. (^∗^ *p <* 0.05, ^∗∗^ *p <* 0.01, ^∗∗∗^ *p <* 0.001).

Additionally, mRNA profiles of signature genes exhibited significant variation across different clinical subgroups. All four mRNA profiles demonstrated enhanced expression in patients with lymph node metastasis (**Figures 9A–D**), but no similar trend was observed in those with the M1 stage, possibly due to the limited sample size (**Supplementary Figures 6A–D**). Additionally, *CTXN1* and *TMEM163* showed marked upregulation in patients with advanced T stages (**Figures 9E–H**). Elderly PTC patients exhibited significantly elevated mRNA levels of *CTXN1*, *MGAT3*, and *TMEM163*. Stratification by age revealed that these elevated expression patterns in older patients were associated with more advanced clinical stages. Immunohistochemical staining was performed to assess the alterations in corresponding protein expression (**Figure 9I–L, N**), and the results revealed that the changes of *ALDH1A3*, *CTXN1*, *MGAT3*, and *TMEM163* protein abundance were consistent with their transcriptional levels. The transcriptome profiling of four metastasis-related biomarkers was reassessed in merged PTMC cohorts (**Figure 9M**). The Comprehensive information regarding the antibodies and experimental conditions used for IHC staining is provided in **Supplementary Table 6**. The inter-rater reliability analysis demonstrated substantial agreement across all signeature markers, with ICC values ranging between 0.857 and 0.908 (**Supplementary Table 7**).

### 3.10 Molecular docking insights into drug sensitivity

**Figure 10:**
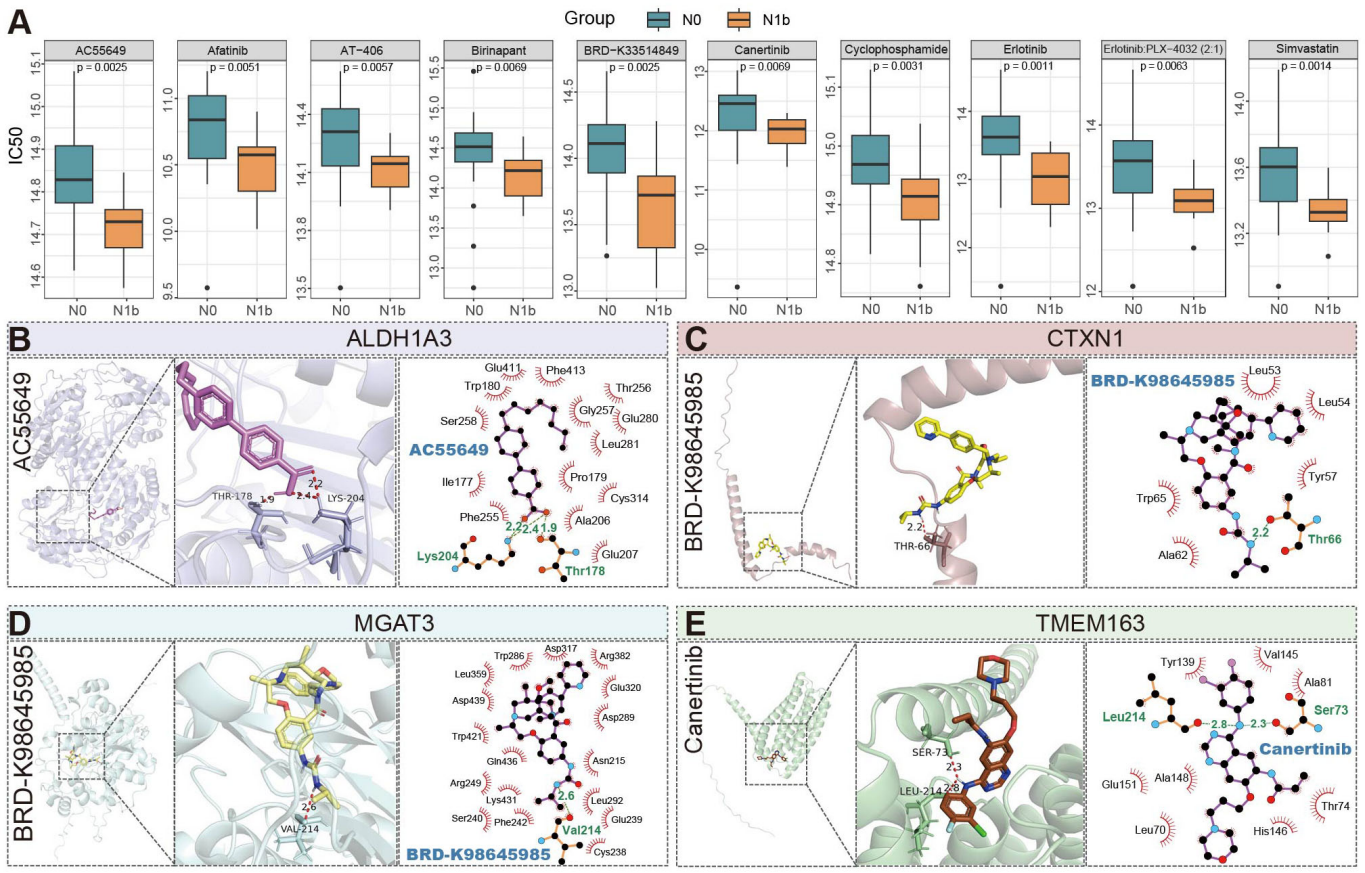
Profiling anticancer drug sensitivity and molecular interactions in PTMC patients stratified by nodal status. **(A)** Top 10 antineoplastic agents with significantly different predicted sensitivities. **(B–E)** Three-dimensional and planar docking conformation results of four proteins with antitumor compounds, with key binding sites and hydrogen bonds (light green) highlighted (PLX4032: Vemurafenib; AT-406: Xevinapant).

Comparative analysis revealed patients with lateral cervical metastases exhibited lower IC_50_ compared to non-metastatic controls (**Figure 10**), indicating heightened drug sensitivity for a range of agents (afatinib, birinapant, AC-55649, AT-406, cyclophosphamide, simvastatin, erlotinib (+vemurafenib), canertinib, BRD-K33514849). Molecular docking simulations demonstrated that BRD-K98645985 formed stable hydrogen bonds with both *MGAT3* and *CTXN1* proteins, with binding free energies (Δ*G*) of -9.5 kcal/mol and -7.2 kcal/mol, respectively. Notably, the *ALDH1A3* protein exhibited consistently strong binding affinities (Δ*G* < -7.0 kcal/mol) with a panel of antitumor agents, including AC55649, afatinib, AT-406, birinapant, canertinib, simvastatin, and erlotinib (**Supplementary Table 8**). The resulting three-dimensional and planar docking conformations revealed favorable molecular complementarity, supporting the presence of a pharmacologically accessible and structurally accommodating binding pocket on *ALDH1A3* (**Supplementary Figure 7**). Findings reveal that *ALDH1A3* may serve as a promising therapeutic target, and its broad compatibility with structurally diverse compounds implies a potentially central role in oncogenic signaling pathways. Furthermore, *TMEM163* also demonstrated appreciable binding affinity with canertinib (Δ*G* = -7.6 kcal/mol), indicating additional potential for targeted therapeutic intervention.

### 3.11 Immune functional and infiltrative alterations with histological confirmation

**Figure 11:**
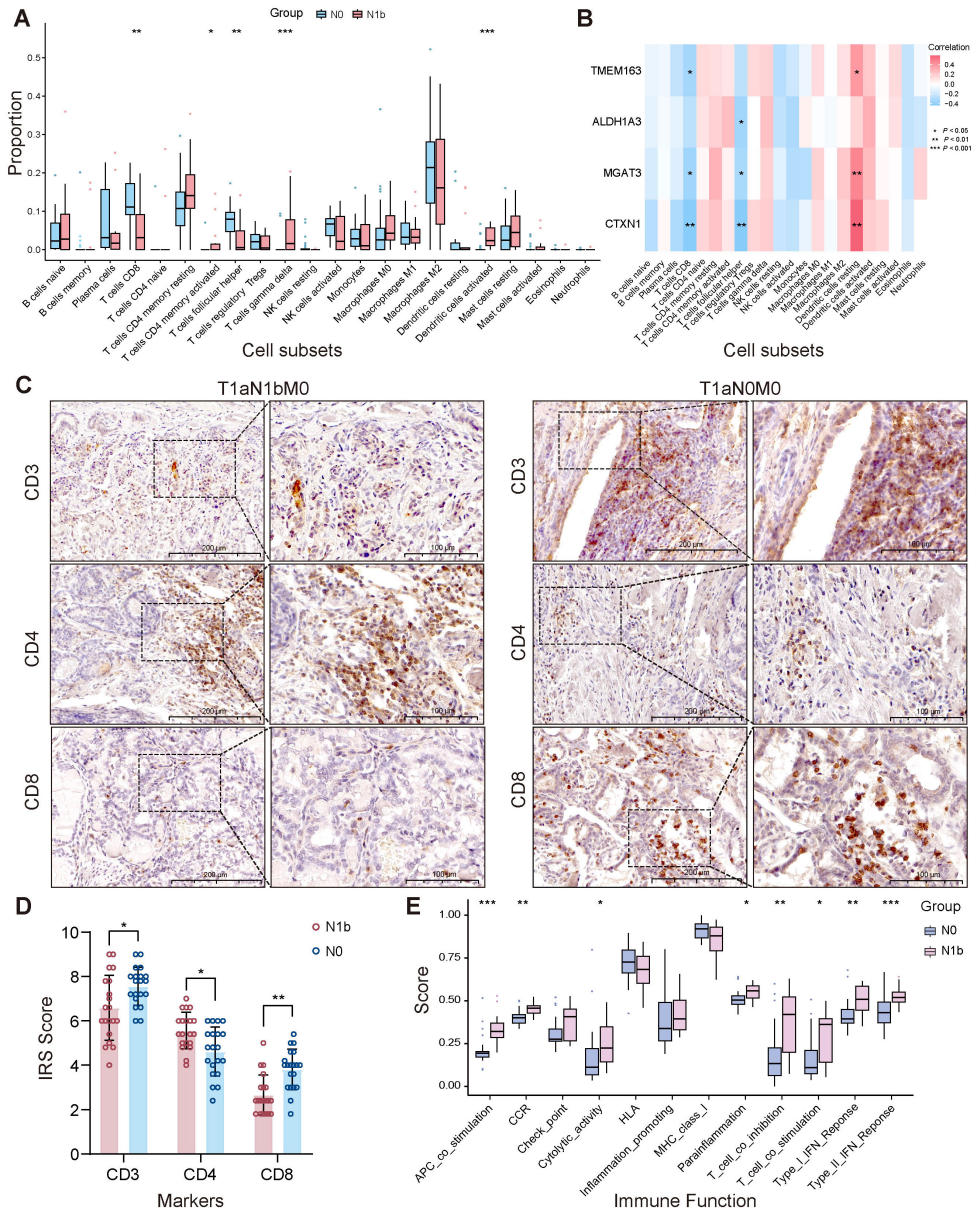
Stage-dependent heterogeneity in tumor-associated immune infiltration. **(A)** Immune infiltration heterogeneity. **(B)** The relationship between signature genes and immune cell populations. **(C, D)** Immunohistochemical validation of distinct lymphocyte subpopulations. **(E)** Immune functional heterogeneity (^∗^ *p <* 0.05, ^∗∗^ *p <* 0.01, ^∗∗∗^ *p <* 0.001).

Grouped immune infiltration analysis of PTMCs utilizing the CIBERSORT approach showed the proportion of 22 types of immune cells (**Supplementary Figure 8A**). Correlation analysis revealed an inverse connection between *γδ* T cells and activated NK cells (*r* = −0.51). In contrast, a significant positive direct correlation was noted between memory B cells and CD4 memory-activated T cells (*r* = 0.9), along with a notable positive relationship with naive CD4 T cells (*r* = 0.88) (**Supplementary Figure 8B**). Comparative analysis of immune cell infiltration revealed distinct patterns. There was a reduced infiltration of follicular helper T cells (Tfh) and CD8+ T cells in the N1b samples. Conversely, CD4 memory-activated T cells, activated dendritic cells, and *γδ* T cells exhibited greater infiltration in the N1b group (**Figure 11A**). Immunohistochemical analysis revealed that, compared to non-metastatic patients, those with lateral cervical lymph node metastasis exhibited reduced T lymphocyte infiltration in the tumor microenvironment, characterized by a marked reduction in CD8+ cytotoxic T cells and an elevation in CD4+ helper T cells (**Figures 11C, D**). Four signature genes exhibited positive correlations with highly infiltrated *γδ* T cells, CD4 memory-activated T cells, and activated dendritic cells in the N1b group and were inversely related to Tfh and CD8+ T cells, which were highly infiltrated in the N0 group (**Figure 11B**). A comparative analysis of immune functions revealed its predominant improvement in the N1b group (**Figure 11E**). Consistently, four signature genes displayed positive correlations with multiple enhanced immune functions in the N1b stage, while exhibiting negative correlations with immune functions upregulated in the N0 stage (**Supplementary Figure 8C**).

## 4 Discussion

Former studies indicated that elevated NLR is a prognostic risk factor in DTC. As the disease progresses, T cell activation and signaling decrease, while circulating neutrophils exhibit marked heterogeneity, with a reduction in mature subsets and an accumulation in immature forms, reflecting tumor-associated inflammation (18). This study comprehensively explored risk factors correlated with lateral neck lymph node metastasis in PTMC by integrating clinical variables and multiple immune-inflammatory indicators. Using both traditional multivariate regression and LASSO regression, we identified potential high-risk factors associated with metastasis, and subsequently established and evaluated them through multiple supervised machine learning approaches combined with cross-validation. Model A, which highlighted NLR as a key risk factor, demonstrated superior performance in terms of stability, predictive accuracy, and discriminative ability. Meanwhile, Model B, characterized by decreased circulating T lymphocytes and increased neutrophils, effectively captured subtle differences in systemic immune responses between PTMC patients with and without advanced lymph node metastasis. Together, both models suggest a decline in antitumor immunity and an increase in tumor-associated pro-inflammatory and pro-metastatic activity in patients with lateral neck lymph node metastases.

To elucidate the transcriptomic landscape of N1b-stage PTC, we applied WGCNA combined with supervised machine learning techniques, developing an MLP model that demonstrated robust performance upon evaluation on an independent test set. Overexpression of signeture genes was intricately linked to the enhancement of cell adhesion molecules and intercellular signaling, potentially driving aberrant KRAS activation, disrupting cholesterol metabolism, and dysregulating MYC target gene expression, thereby facilitating PTMC metastasis. Genomic analysis revealed hypomethylation of *CTXN1*, *MGAT3*, and *TMEM163* genes as a key mechanism promoting metastatic progression. Previous studies have identified inactivating mutations in *MGAT3* (E320A, R382A, H418A) that impair protein function (38). However, only one N1-stage patient in our study harbored the G16D missense mutation. The *MGAT3* p.G16D variant is not currently annotated as a known driver or pathogenic mutation, and its functional implications—particularly in relation to PTMC metastasis—warrant further investigation. Furthermore, the partial deletions of signature genes exhibited no variation across different N stages, and no amplifications were observed in the metastatic group, indicating that their CNAs exert minimal influence on metastasis in this context.

*ALDH1A3*, encoding dehydrogenase family one member A3, is crucial in the oxidation of aldehydes and has been investigated as a potential prognostic biomarker in multiple carcinomas (39–42). Tumor cells with high ALDH expression in PTC tissue exhibit distinct stem-like characteristics and the ability to reinitiate serially transplantable tumors (recapitulate the metastatic behavior of parental tumors) (43). *MGAT3* encodes *β*1,4-N-acetylglucosaminyltransferase III to transfer N-acetylglucosamine (GlcNAc) from UDP-GlcNAc to a double-stranded sugar chain, generating *β*1,4-GlcNAc with a bisecting structure. Its effects on growth, adhesion, invasion, and metastasis can potentially vary due to modifying distinct cell surface proteins. MALDI-TOF-MS evidence has demonstrated increased bisected glycans in PTC tissue compared to normal thyroid (44). However, the specific bisected GlcNAc-modified proteins affecting migration of PTMC require further analysis through mass spectrometry-based investigations and fundamental experiments. Notably, thyroglobulin antibodies (TgAbs) undergo bisected glycosylation, and the removal of glycans abolishes the antibody-dependent cellular cytotoxicity (ADCC) process (45, 46), suggesting abnormal expression of *MGAT3* may influence PTC progression by modulating immune responses. *CTXN1* encodes Cortexin-1, a neuropeptide involved in the development and functional regulation of cortical neurons, including synapse formation, neuronal migration, as well as signal transduction. Knocking out *CTXN1* in GL261 glioma cells in mice reduced tumor burden, improved survival rates, and enhanced infiltration of CD8+ T cells (47). It is highly expressed in breast cancer, and further investigations are necessary to elucidate its potential role in PTMC metastasis (48). *TMEM163* encodes transmembrane protein 163, which is predominantly expressed in myelinating oligodendrocytes of the central nervous system. It has been characterized as a zinc-binding protein involved in intracellular zinc transport (49), which is instrumental in the clearance of reactive oxygen species (ROS) and is essential for the immune system (50, 51). The overexpression of *TMEM163*, induced by the oncogene Src, has been demonstrated to facilitate the migration of transformed tumor cells (52).

Accumulating evidence indicates that the immune landscape of PTC evolves with tumor progression. In N1-stage tumors, a notable decline in intratumoral CD8+ T cell infiltration is observed, together with heightened levels of resting and activated DCs, *γδ* T cells, and resting memory CD4+ T cells (53). Importantly, decreased infiltration of CD8+ T cells in the tumor milieu has been consistently associated with adverse survival outcomes in PTC (54). In contrast to previously reported findings in large-volume PTC, our study revealed distinct immune infiltration patterns in PTMC associated with LLNM (N1b stage). Specifically, compared to N0 tumors, N1b tumors exhibited reduced infiltration of cytotoxic CD8+ T lymphocytes and Tfh cells, alongside increased infiltration of *γδ* T cells, activated CD4+ memory T cells, and activated dendritic cells. These findings suggest that the attenuation of cytotoxic CD8+ T cells and humoral-supportive Tfh cells in N1b-stage PTC may impair tumor-specific immune clearance, thereby promoting immune evasion and metastatic progression. DCs, as antigen-presenting cells, are key players in both immune defense and immune evasion mechanisms in thyroid cancer; the increased proportion of activated DCs in N1b tumors may enhance the priming of naive T cells and their progression toward effector phenotypes, thereby enhancing tumor recognition. Concurrently, the increased presence of activated CD4+ memory T cells in the TME reflects strengthened T cell memory function, facilitating the generation of antigen-specific CD4+ effector T cells and amplifying helper immune responses to recruit additional effector cells. *γδ* T cells cells function in both innate and adaptive immunity, directly targeting tumor cells independent of MHC presentation and mediating antitumor activity through NK cell receptors, ADCC, and cytokine secretion (e.g., IFN-*γ*, TNF-*α*), although their efficacy is often limited by complex tumor immune evasion mechanisms (55). The increased infiltration of *γδ* T cells in metastatic PTMC likely represents an augmented immune surveillance response, but under the immunosuppressive tumor microenvironment, these cells may polarize toward pro-tumorigenic subsets, such as IL-17-producing *γδ* T cells, thereby promoting metastasis. Given the immunologically “cold” nature of early metastatic PTMC, emerging CAR-*γδ* T cell therapies hold promising potential as innovative treatment strategies.

This study reveals alterations in circulating immune indicators—particularly NLR, lymphocytes, and neutrophils-reflect a systemic antitumor immune response in highly metastatic PTMC. Multi-omics integration identified key molecular markers (*ALDH1A3*, *CTXN1*, *MGAT3*, and *TMEM163*) linked to cell adhesion, migration, metabolism, and immune modulation. Notably, advanced-stage PTMC is characterized by reduced infiltration of CD8+ T and Tfh cells, alongside increased *γδ* T cells, activated DCs, and activated memory CD4+ T cells within the TME. Although our multilevel integrated analysis offers new insights into the clinical and molecular landscape, systemic immunity, and immune microenvironment of advanced PTC, further validation through large-scale clinical multi-omics datasets and mechanistic investigations is warranted to substantiate and extend these findings.

## Data Availability

The original contributions presented in the study are included in the article/**Supplementary Material**. Further inquiries can be directed to the corresponding author.
