# Supplementary material for: Multi-omics analysis and metastasis risk factor prediction in N1b stage PTMC: insights into immune infiltration and therapeutic implications
Source: Front Immunol. 2025 Sep 3;16:1620085. doi: 10.3389/fimmu.2025.1620085 (PMC12444076; doi:10.3389/fimmu.2025.1620085)
Supplement: Supplementary file 1 [file DataSheet1.docx]

Supplementary Materials

Multi-Omics Analysis and Metastasis Risk Factor Prediction in N1b Stage PTMC: Insights into Immune Infiltration and Therapeutic Implications

Hao Dai, Qian Zhao, Wanli Ren, Qian Chen, Bei Pei, Wenyan Wang, Zhiqian Liu, Zhihan Liu, Jinzi Guo, Yuan Shao, Xiang Li, Yanxia Bai*

*** Correspondence:** Yanxia Bai: yanxiab@xjtu.edu.cn

# Supplementary Figures

#
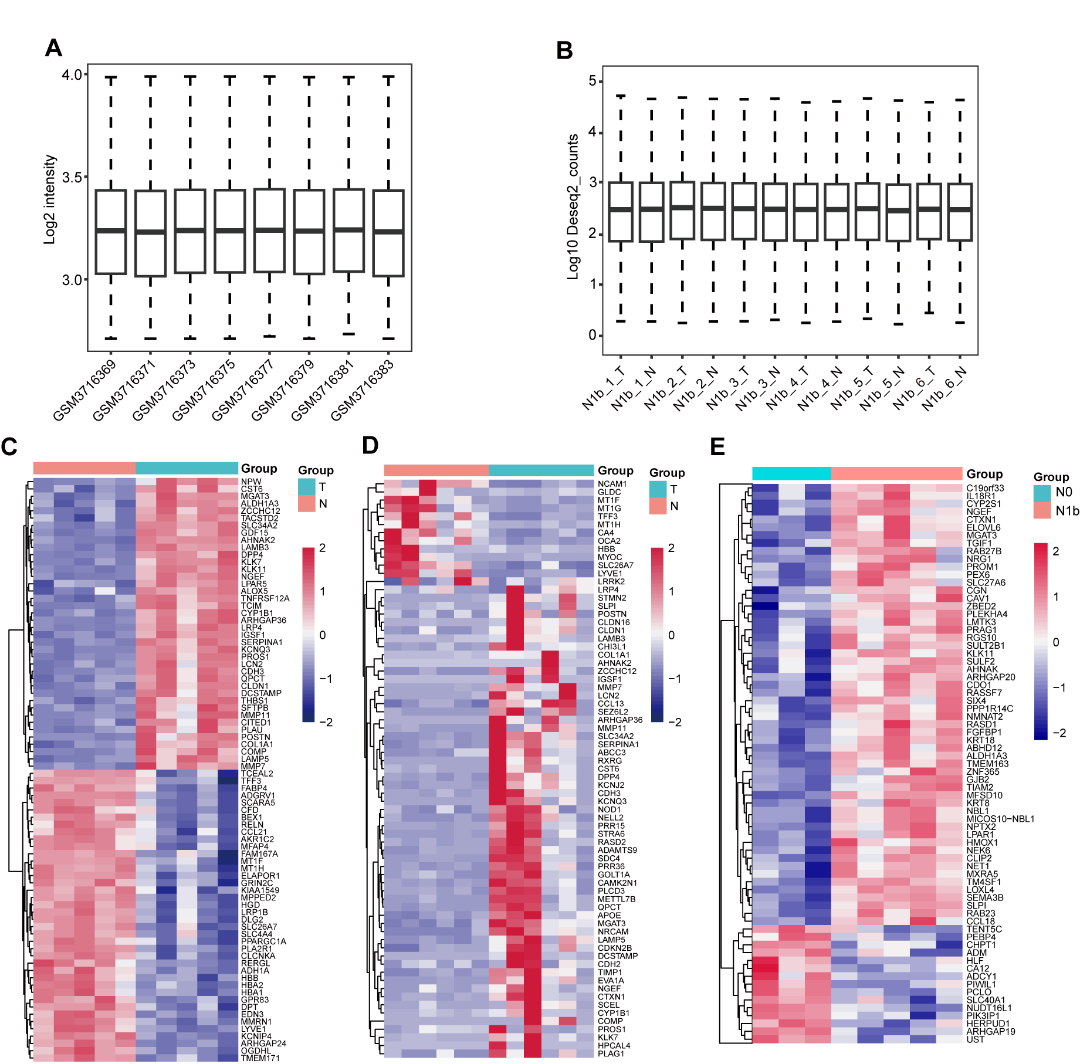


**Supplementary Figure 1.** The transcriptional profiling comparison. Quality assessment and standardization of the (A) GSE129562 dataset and (B) the papillary thyroid microcarcinoma transcriptome atlas (PTMTA) dataset. Heatmap of major DEGs between N1b-stage PTMC and matched adjacent thyroid tissues in the (C) GSE129562 and (D) PTMTA datasets. (E) DEGs between N1b-stage and N0-stage PTMC in the GSE129562 dataset.

**
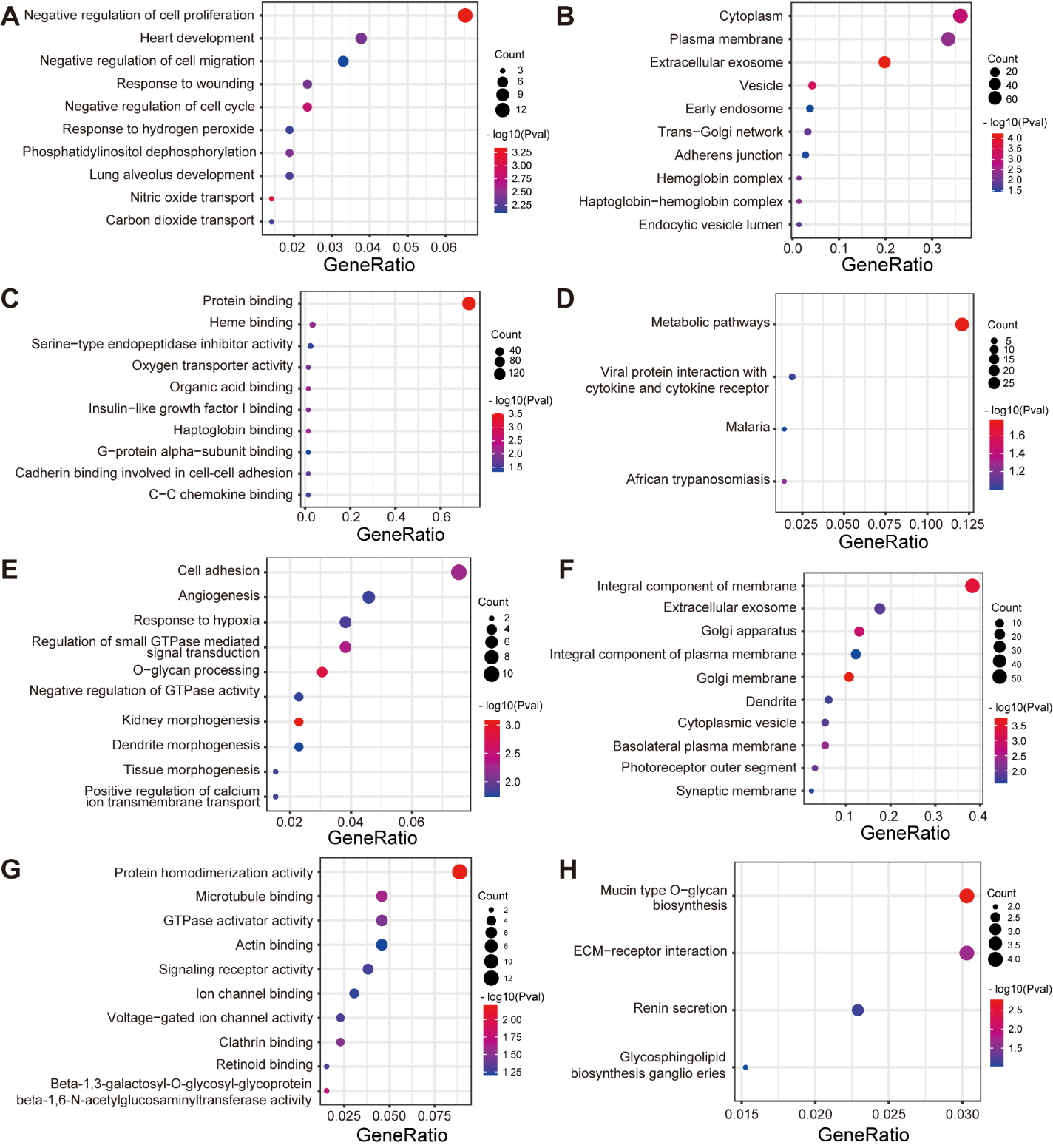
**

**Supplementary Figure 2.** Bubble plots illustrating functional enrichment analysis. Enrichment results for genes in the pink module: (A) Biological Processes (BP), (B) Cellular Components (CC), (C) Molecular Functions (MF), and (D) KEGG pathways. Enrichment results for biomarkers in the green-yellow module: (E) BP, (F) CC, (G) MF, and (H) KEGG pathways.

**
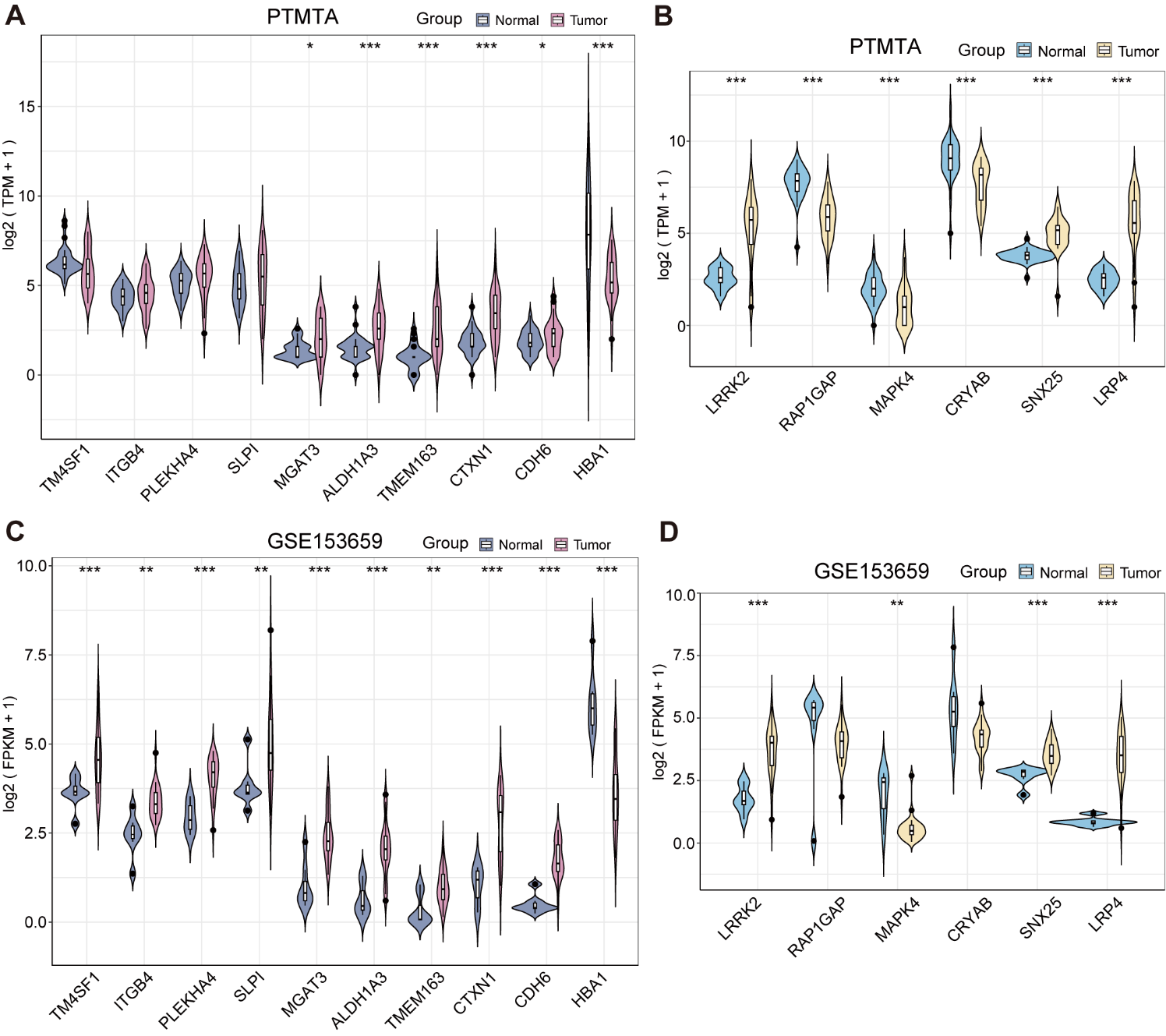
**

**Supplementary Figure 3.** Validation of the transcriptional profiles of candidate genes from (A) the pink module in TCGA-PTMC and (C) the GSE153659 cohort. Transcriptional profiling validation of the candidate genes from the green-yellow module in (B) TCGA-PTMC and (D) GSE153659 cohort. (**p* < 0.05, ** *p* < 0.01, *** *p* < 0.001).


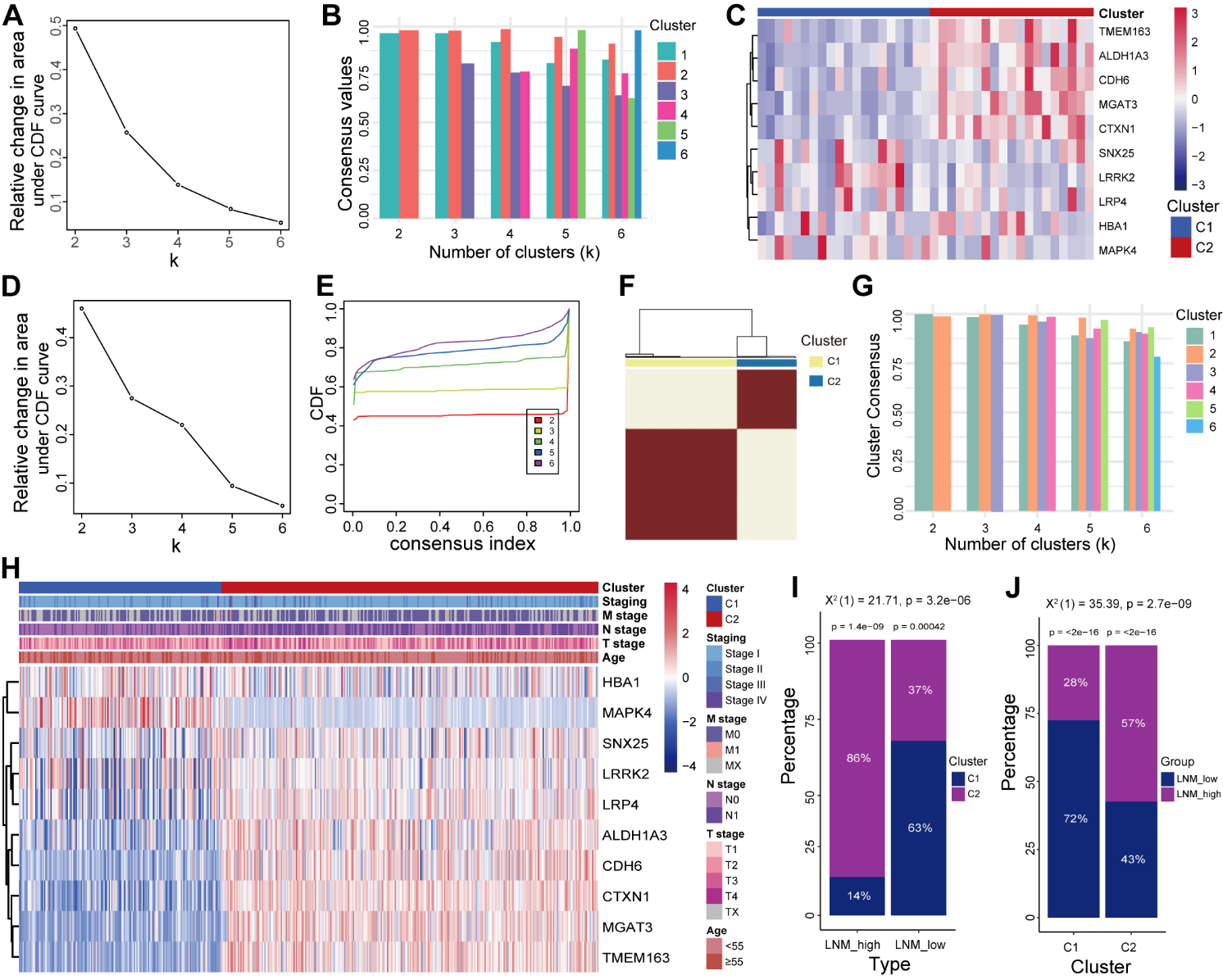


**Supplementary Figure 4.** Validation of cluster typing of differential candidate genes in (A-C) the merged PTMC cohort and (D-J) the large-volume TCGA-PTC cohort. (A, D) The Delta area plot illustrates the region's relative variation beneath the CDF curve (k = 2 - 6). (E) CDF trajectory plot for consensus clustering (k = 2 - 6). (F) Consensus matrix plot (k = 2). (B, G) Cluster-consensus plot for different k values. (C, H) Heatmap of differentially expressed metastasis-related genes between the two clusters. (I) Distribution of patients with aggressive versus indolent metastasis in C1 and C2 clusters of the large-volume PTC cohort. (J) Proportion of patients with varying N stages within the C1 and C2 clusters.


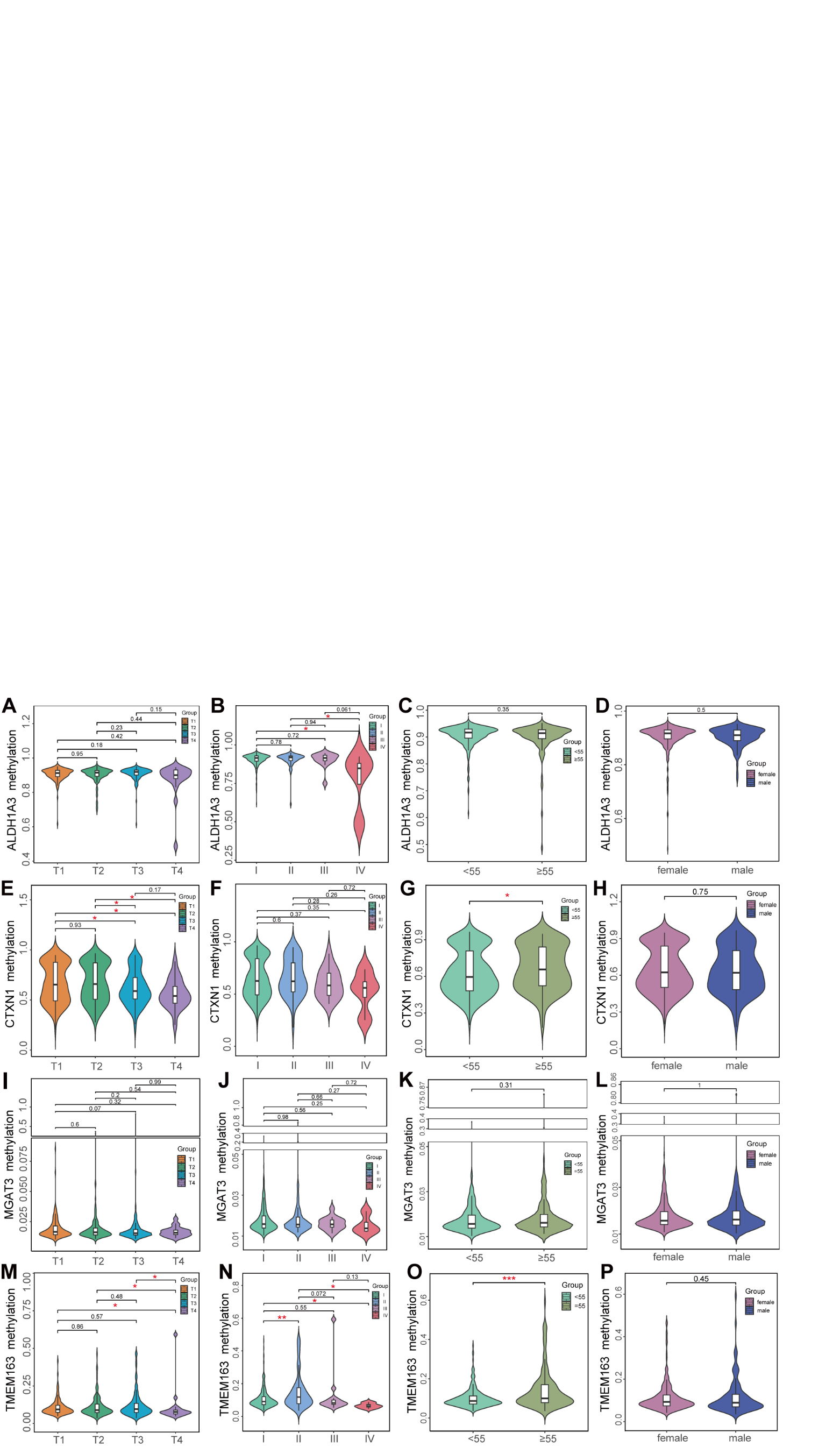


**Supplementary Figure 5.** Comparison of methylation levels of (A-D) *ALDH1A3*, (E-H) *CTXN1*, (I-L) *MGAT3*, and (M-P) *TMEM163* across clinicopathological subgroups in the PTC cohort.


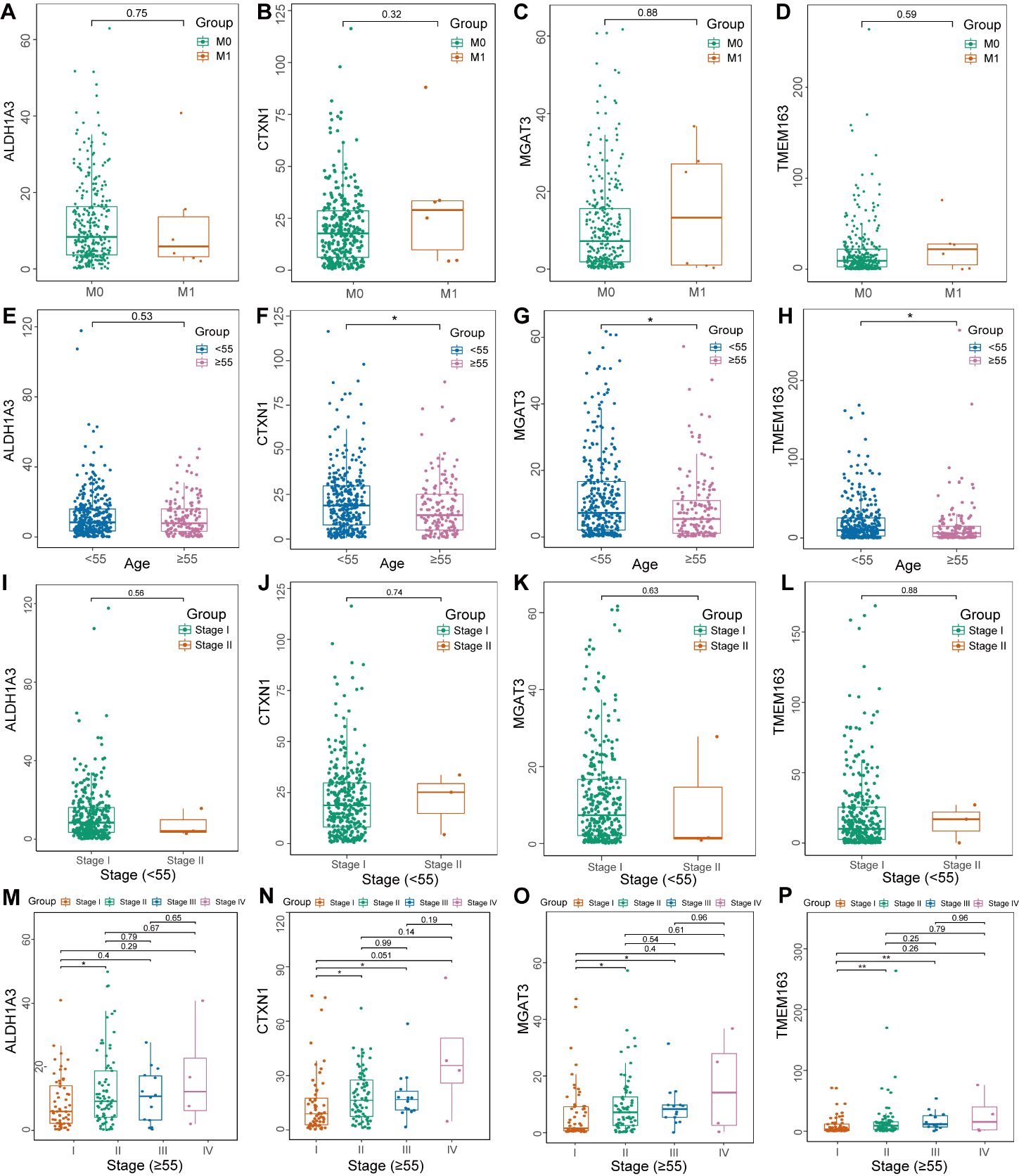


**Supplementary Figure 6.** Transcriptional discrepancies of signature genes across (A-D) M stages and (E-H) age groups. Age-stratified transcriptional differences across distinct clinical stages in TCGA-PTC populations, with (I-L) < 55 years and (M-P) ≥ 55 years shown in panels (**p* < 0.05, ** *p* < 0.01).


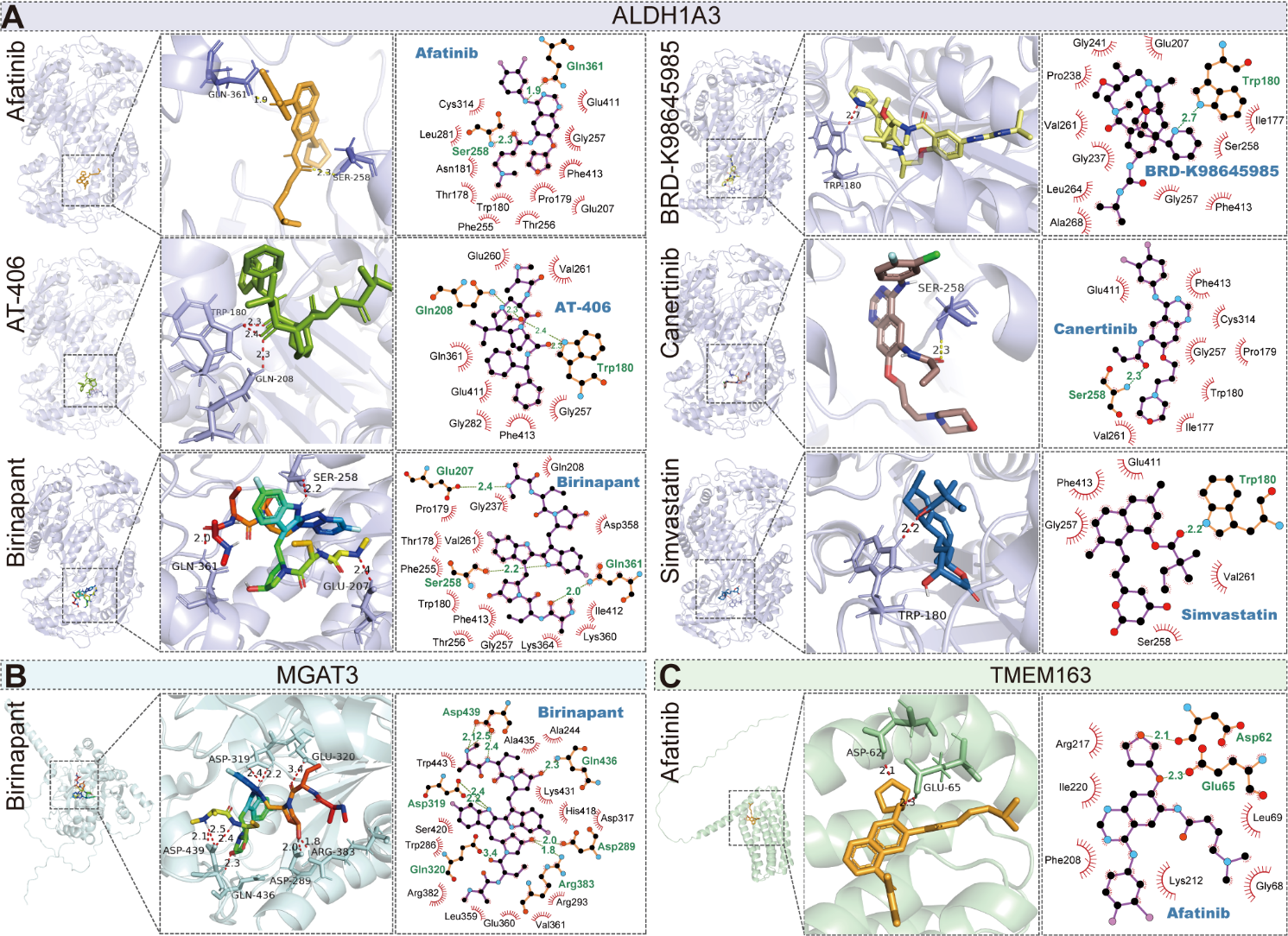


**Supplementary Figure 7.** Representative molecular docking conformations illustrating the binding interactions between target proteins (A) *ALDH1A3*, (B) *MGAT3*, and (C) *TMEM163* and their respective candidate antitumor compounds.


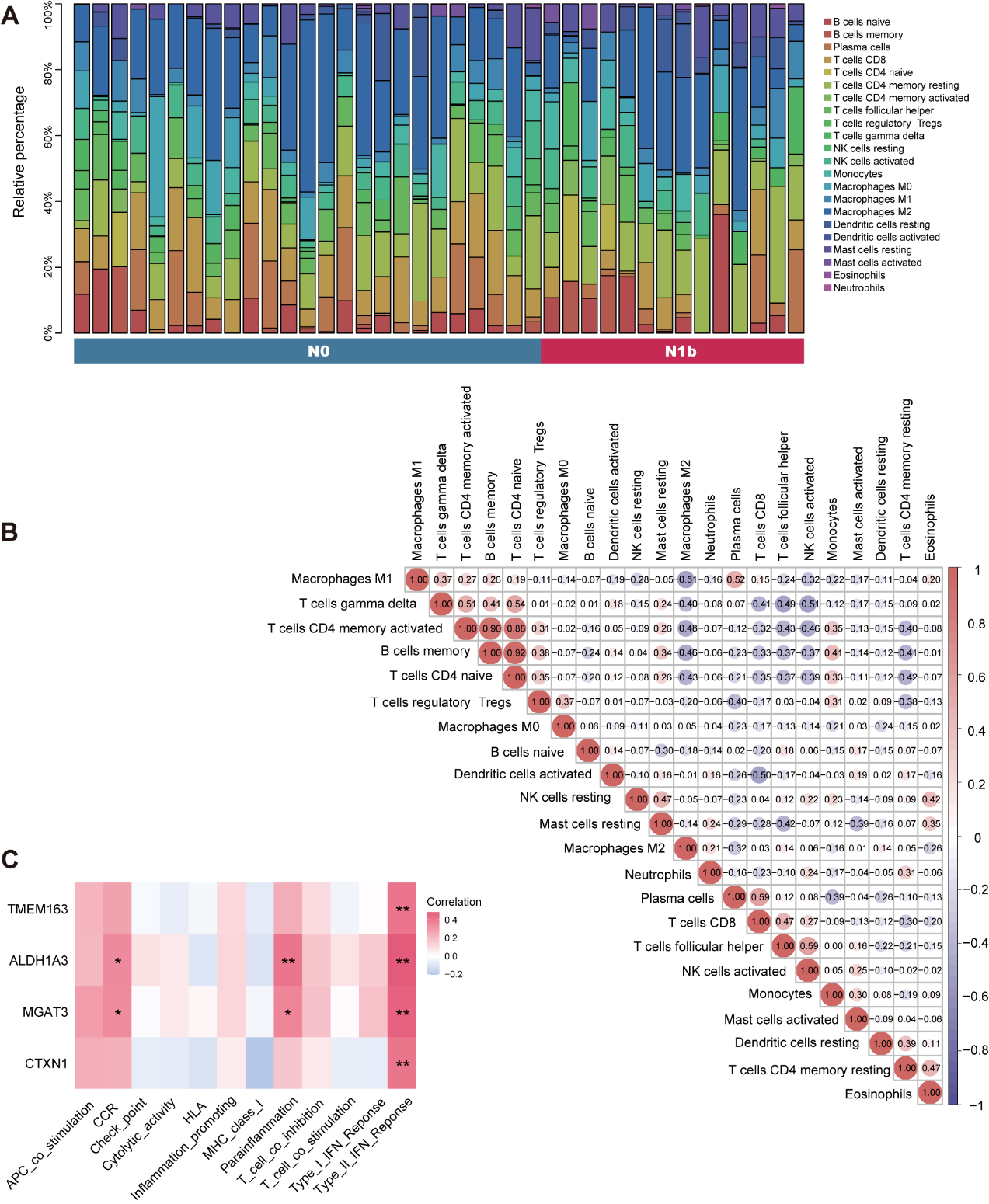


**Supplementary Figure 8.** Differential immune cell infiltration between N stages. (A) Grouped stacked plots visually depicted the relative percentage of immune cells. (B) Heatmap illustrating the intercorrelations among 22 immune cell types. (C) The association between signature genes and immune cell populations (∗ *p* < 0.05, ∗∗ *p* < 0.01).

# Supplementary Tables

**Supplementary Table 1** Baseline clinical and hematological characteristics of the PTMC cohort.

| Characteristic | N0 (N=490) | N1b (N=148) | p |
| --- | --- | --- | --- |
| Age, median (IQR) | 46 (38 - 54) | 38 (32 - 48) | <.001^*^ |
| Gender, *n%* |  |  |  |
| Female | 394 (80.4%) | 104 (70.3%) | .012 |
| Male | 96 (19.6%) | 44 (29.7%) |  |
| Lymphocytes (10^9^/L), median (IQR) | 1.8 (1.6 - 2.2) | 1.8 (1.5 - 2.1) | .029^*^ |
| Monocytes (10^9^/L), median (IQR) | 0.3 (0.2 - 0.4) | 0.3 (0.2 - 0.4) | .660^*^ |
| Neutrophils (10^9^/L), median (IQR) | 3.3 (2.5 - 4.0) | 3.4 (2.9 - 4.5) | .004^*^ |
| Platelet (10^9^/L), median (IQR) | 221.5 (187 - 259) | 216.5 (184 - 268) | .965^*^ |
| NLR, median (IQR) | 1.7 (1.4 - 2.2) | 2.0 (1.6 - 2.6) | <.001^*^ |
| PLR, median (IQR) | 120.7 (97.8 - 146) | 123.1 (103.1 - 157) | .050^*^ |
| LMR, median (IQR) | 6.2 (5.0 - 7.6) | 5.9 (4.7 - 7.3) | .153^*^ |
| SII, median (IQR) | 384.6 (283.8 - 506.9) | 452.9 (316.1 -648.8) | <.001^*^ |
| ETE, *n* % |  |  |  |
| No | 228 (46.5%) | 22 (14.9%) | <.001 |
| Yes | 262 (53.5%) | 126 (85.1%) |  |
| HT, *n* % |  |  |  |
| No | 392 (80%) | 121 (81.8%) | .724 |
| Yes | 98 (20%) | 27 (18.2%) |  |
| NG, *n* % |  |  |  |
| No | 406 (82.9%) | 122 (82.4%) | 1.000 |
| Yes | 84 (17.1%) | 26 (17.6%) |  |
| Multifocality, *n* % |  |  |  |
| No | 363 (74.1%) | 49 (33.1%) | <.001 |
| Yes | 127 (25.9%) | 99 (66.9%) |  |
| Lesion location, *n* % |  |  |  |
| Bilateral | 90 (18.4%) | 61 (41.2%) | <.001 |
| Bilateral-isthmus | 5 (1%) | 1 (0.7%) |  |
| Isthmus | 3 (0.6%) | 1 (0.7%) |  |
| Unilateral | 373 (76.1%) | 75 (50.7%) |  |
| Unilateral-isthmus | 19 (3.9%) | 10 (6.8%) |  |

*Wilcoxon rank sum test. IQR, interquartile range; ETE, Extrathyroidal Extension; HT, Hashimoto's thyroiditis; NG, Nodular goiter.

**Supplementary** **Table 2** Cross-validated performance metrics of eight models on the training set.

| Model | AUC (95%CI) | Accuracy | Brier Score | Sensitivity | Specificity | F1 Score | Youden Index |
| --- | --- | --- | --- | --- | --- | --- | --- |
| AdaBoost | 0.818 (0.727 - 0.872) | 0.792 | 0.135 | 0.915 | 0.385 | 0.871 | 0.300 |
| Decision-tree | 0.752 (0.653 - 0.864) | 0.792 | 0.149 | 0.921 | 0.365 | 0.872 | 0.287 |
| KNN | 0.803 (0.727 - 0.887) | 0.781 | 0.152 | 0.886 | 0.433 | 0.861 | 0.319 |
| Logistic | 0.806 (0.690 - 0.868) | 0.828 | 0.125 | 0.945 | 0.442 | 0.894 | 0.387 |
| MLP | 0.754 (0.632 - 0.835) | 0.803 | 0.166 | 0.921 | 0.413 | 0.878 | 0.335 |
| Random-forest | 0.797 (0.710 - 0.908) | 0.796 | 0.143 | 0.933 | 0.356 | 0.877 | 0.289 |
| SVM | 0.787 (0.713 - 0.921) | 0.819 | 0.135 | 0.968 | 0.365 | 0.896 | 0.333 |
| XGBoost | 0.803 (0.726 - 0.845) | 0.785 | 0.167 | 0.892 | 0.433 | 0.864 | 0.325 |

**Supplementary Table 3** Evaluation metrics of eight models on the test set.

| Model | AUC (95%CI) | Accuracy | Brier Score | Sensitivity | Specificity | F1 Score | Youden Index |
| --- | --- | --- | --- | --- | --- | --- | --- |
| Adaboost | 0.835 (0.761−0.910) | 0.806 | 0.128 | 0.857 | 0.636 | 0.872 | 0.494 |
| KNN | 0.858 (0.795−0.922) | 0.827 | 0.127 | 0.891 | 0.614 | 0.888 | 0.505 |
| Logistic | 0.852 (0.782−0.922) | 0.853 | 0.118 | 0.918 | 0.636 | 0.906 | 0.555 |
| MLP | 0.846 (0.775−0.917) | 0.838 | 0.156 | 0.925 | 0.545 | 0.898 | 0.471 |
| Random-forest | 0.837 (0.762−0.913) | 0.859 | 0.109 | 0.952 | 0.545 | 0.912 | 0.498 |
| SVM | 0.848 (0.771−0.925) | 0.869 | 0.104 | 0.980 | 0.500 | 0.920 | 0.480 |
| Decision-tree | 0.810 (0.731−0.889) | 0.874 | 0.104 | 0.980 | 0.523 | 0.923 | 0.502 |
| XGBoost | 0.823 (0.740−0.905) | 0.843 | 0.121 | 0.912 | 0.614 | 0.899 | 0.525 |

**Supplementary Table 4** Clinical parameters of paired N1b stage PTMCs.

| **Sample ID** | **Gender** | **Age** | **pT** | **pN** | **pM** | **AJCC(8th-edition) Pathologic Stage** | **Histological type** | **Mutation** | **TI-RADS** | **Tumor size (greatest diameter, cm)** |
| --- | --- | --- | --- | --- | --- | --- | --- | --- | --- | --- |
| B1-Tumor | Female | 32 | T1a | N1b | M0 | I | Classical | -- | 4 | 0.8 |
| B1-Normal | Female | 32 | -- | -- | -- | -- | -- | -- | -- | -- |
| B2-Tumor | Male | 43 | T1a | N1b | M0 | I | Classical | -- | 4 | 1 |
| B2-Normal | Male | 43 | -- | -- | -- | -- | -- | -- | -- | -- |
| B3-Tumor | Female | 36 | T1a | N1b | M0 | I | Classical | *BRAF* | 4 | 0.7 |
| B3-Normal | Female | 36 | -- | -- | -- | -- | -- | -- | -- | -- |
| B4-Tumor | Female | 40 | T1a | N1b | M0 | I | Classical | -- | 4 | 0.9 |
| B4-Normal | Female | 40 | -- | -- | -- | -- | -- | -- | -- | -- |
| B5-Tumor | Male | 41 | T1a | N1b | M0 | I | Classical | -- | 4 | 1 |
| B5-Normal | Male | 41 | -- | -- | -- | -- | -- | -- | -- | -- |
| B6-Tumor | Female | 39 | T1a | N1b | M0 | I | Classical | -- | 4 | 0.9 |
| B6-Normal | Female | 39 | -- | -- | -- | -- | -- |  | -- | -- |

**Supplementary Table 5** Clinical data of the TCGA-PTMC dataset.

| **Tumor Sample Barcode** | **Gender** | **Age** | **pT** | **pN** | **pM** | **AJCC(8th-edition) Pathologic Stage** | **Histological type** | **Driver Mutation** | **Tumor size (greatest diameter, cm)** |
| --- | --- | --- | --- | --- | --- | --- | --- | --- | --- |
| TCGA-DJ-A3UX-01 | Female | 46 | T1 | N0 | M0 | I | Classical | *BRAF* | 0.5 |
| TCGA-BJ-A2N8-01 | Female | 30 | T1b | N0 | M0 | I | Classical | *NRAS* | 1 |
| TCGA-DJ-A3VL-01 | Male | 38 | T1a | N0 | M0 | I | Follicular variant | *RASA* | 0.6 |
| TCGA-DJ-A2PO-01 | Male | 54 | T1a | N0 | M0 | I | Classical | *-* | 0.6 |
| TCGA-DJ-A1QN-01 | Female | 42 | T1b | N0 | M0 | I | Classical | *BRAF* | 0.7 |
| TCGA-DJ-A1QM-01 | Male | 42 | T1a | N0 | M0 | I | Follicular variant | *BRAF* | 0.7 |
| TCGA-E8-A415-01 | Female | 39 | T1a | N1b | M0 | I | Classical | *BRAF* | 1 |
| TCGA-EM-A3FJ-01 | Female | 24 | T1a | N1b | MX | I | Classical | *BRAF* | 1 |
| TCGA-ET-A3DS-01 | Female | 33 | T1 | N0 | MX | I | Classical | *-* | 0.8 |
| TCGA-BJ-A45E-01 | Female | 46 | T1a | N0 | M0 | I | Classical | *-* | 0.5 |
| TCGA-ET-A25P-01 | Female | 24 | T1 | N0 | MX | I | Classical | *-* | 0.6 |
| TCGA-FY-A40K-01 | Female | 46 | T1a | N0 | MX | I | Classical | *BRAF* | 1 |
| TCGA-DJ-A3UN-01 | Female | 51 | T1b | N0 | M0 | I | Classical | *BRAF* | 1 |
| TCGA-DJ-A3UZ-01 | Female | 70 | T3 | N0 | M0 | II | Classical | *-* | 0.8 |
| TCGA-ET-A39S-01 | Female | 27 | T1 | N0 | MX | I | Classical | *BRAF* | 1 |
| TCGA-DJ-A4V4-01 | Female | 48 | T1a | N0 | M0 | I | Columnar cell | *BRAF* | 0.9 |
| TCGA-DJ-A3UT-01 | Female | 43 | T1a | N0 | M0 | I | Follicular variant | *NRAS* | 0.9 |
| TCGA-E8-A434-01 | Female | 56 | T1a | N0 | M0 | I | Classical | *BRAF* | 0.7 |
| TCGA-FY-A3R9-01 | Female | 66 | T1a | N0 | MX | I | Classical | *NRAS* | 0.7 |
| TCGA-ET-A3DQ-01 | Female | 43 | T1 | N0 | MX | I | Follicular variant | *-* | 1 |
| TCGA-FY-A40N-01 | Female | 55 | T1a | N0 | MX | I | Follicular variant | *-* | 0.9 |
| TCGA-DJ-A4UT-01 | Female | 32 | T1a | N0 | M0 | I | Classical | *-* | 1 |
| TCGA-DJ-A3VF-01 | Female | 60 | T3 | N1b | M0 | II | Columnar cell | *BRAF* | 1 |
| TCGA-DJ-A3VK-01 | Male | 63 | T3 | N0 | M0 | II | Follicular variant | *-* | 1 |
| TCGA-BJ-A45C-01 | male | 78 | T3 | N0 | M0 | II | Classical | *-* | 1 |

**Supplementary Table 6** Antibody panel and experimental parameters.

| **Compound** | **Catalog No.** | **Source** | **Host Species** | **Dilution** | **Retrieval Method** |
| --- | --- | --- | --- | --- | --- |
| *ALDH1A3* | [25167-1-AP](https://ptgcn.com/products/ALDH1A3-Antibody-25167-1-AP.htm) | Proteintech | Rabbit | 1:300 | Tris-EDTA, pH 9.0 |
| *MGAT3* | 17869-1-AP | Proteintech | Rabbit | 1:200 | Tris-EDTA, pH 9.0 |
| *CTXN1* | PA5-53472 | Invitrogen | Rabbit | 1:400 | Tris-EDTA, pH 9.0 |
| *TMEM163* | PA5-114329 | Invitrogen | Rabbit | 1:50 | Tris-EDTA, pH 9.0 |
| *CD3* | YM8134 | Immunoway | Rabbit | 1:500 | Citrate, pH 6.0 |
| *CD8α* | YM8067 | Immunoway | Rabbit | 1:500 | Citrate, pH 6.0 |
| *CD4* | YM8254 | Immunoway | Rabbit | 1:500 | Citrate, pH 6.0 |

**Supplementary Table 7** Inter-rater reliability of IHC scoring.

| **Marker** | **ICC**  **(Average Measures)** | **95% CI** | ***p* Value** |
| --- | --- | --- | --- |
| *ALDH1A3* | [0.873](https://ptgcn.com/products/ALDH1A3-Antibody-25167-1-AP.htm) | (0.767-0.935) | <.001 |
| *MGAT3* | 0.908 | (0.831-0.953) | <.001 |
| *CTXN1* | 0.882 | (0.783-0.940) | <.001 |
| *TMEM163* | 0.857 | (0.738-0.927) | <.001 |
| *CD3* | 0.952 | (0.918-0.973) | <.001 |
| *CD8α* | 0.938 | (0.895-0.965 | <.001 |
| *CD4* | 0.928 | (0.879-0.959) | <.001 |

**Supplementary** **Table 8** Minimum binding energies of the small molecular anticancer agents with multiple target proteins ^a^.

| **Compound** | **ALDH1A3 (kcal/mol)** | **CTXN1 (kcal/mol)** | **MGAT3 (kcal/mol)** | **TMEM163 (kcal/mol)** |
| --- | --- | --- | --- | --- |
| AC55649 | -9.7 | - | -7.5 | - |
| Afatinib | -8.6 | - | -7.8 | -7.3 |
| AT406 | -8.1 | - | -7.9 | - |
| Birinapant | -8.5 | - | -8.7 | -7.3 |
| BRD-K98645985 | -8.7 | -7.2 | -9.5 | - |
| Canertinib | -8.9 | - | -7.6 | -7.7 |
| Erlotinib | -7.4 | - | -7.9 | - |
| Simvastatin | -8.2 | - | -7.8 | - |
| Cyclophosphamide | - | - | - | - |

^a^ Molecular docking performed using AutoDock Vina (v1.1.2).

# Supplementary Methods

**cDNA Library Construction and QC Metrics for RNA-Seq**

We employed a NanoDrop-1000 spectrophotometer (Wilmington, DE, USA) to detect its quantity and purity. The samples were validated and ensured for RNA integrity greater than 7.0 using the Agilent 2100 Bioanalyzer. After removing ribosomal RNA employing TruSeq Stranded Total Library Prep Kit (Illumina, San Diego, CA, USA), a cDNA library with a mean insert’s length of 300±50 base pairs were generated based on ∼1 μg of total RNA. The cDNA library was subjected to sequencing utilizing the Illumina NovaSeq 6000 platform, yielding 150 bp paired-end reads. Cutadapt (v2.6) was used to trim adapters and filter out low-quality reads, ensuring data integrity and reducing noise^[1]^. Then, sequencing reads were mapped onto the GRCh38 reference genome utilizing GENCODE v23 annotation using HISAT2 (v.2.1.0)^[2]^.

**Co-Expression Module Identification and Clustering**

After excluding outlier samples, we defined a value of 0.80 for the scale-free topology fitting index $\text{R}^{2}$ to assess the existence of a scale-free topology and selected the optimum soft threshold utilizing the pickSoftThreshold. The adjacency function (); with denoting the adjacency matrix connecting node i to node j, : the positive value of the correlation metric between node $\text{i}$and node $\text{j}$) computes the adjacency matrix from the correlation matrix. The adjacency matrix was then transformed into an unsigned topological overlap matrix. It was then subjected to average chained hierarchical clustering, enabling the classification of genes into distinct coexpression modules using the hclust function.

**MLP-Based Prediction and Evaluation**

The MLP model comprises input, hidden, and output layers. The input value $\text{α}$ of each neuron in the hidden layers is computed as , where and are respectively defined as the weight and score of the $\text{i}$-th gene. Nonlinear activation functions are used to achieve the nonlinear transformation of input information. The relative importance of the input variables in the neural network was visualized utilizing the "NeuralNetTools" (v.1.5.3) package. The "pROC" package facilitated the generation of receiver operating characteristic (ROC) curve for the training set.

**Gene Significance and Intramodular Connectivity**

The average GS for all modules is, where $\text{P}$represents the correlation between individual gene profiles and metastasis within the particular module. Module membership, denoted as , where $\text{E}^{\text{(q)}}$ denotes the MEs for module $\text{q}$ and is the profile of gene$\text{i}$, was employed to indicate the correlation between gene profile and MEs.

[1] Martin M.Cutadapt removes adapter sequences from high-throughput sequencing reads[J].EMBnet. journal,2011, 17 (1): 10-12.

[2] Pertea M, Pertea G M, Antonescu C M, et al.StringTie enables improved reconstruction of a transcriptome from RNA-seq reads[J].Nat Biotechnol,2015, 33 (3): 290-5.
